# Effect of Ag modification on TiO_2_ and melem/g-C_3_N_4_ composite on photocatalytic performances

**DOI:** 10.1038/s41598-023-32094-6

**Published:** 2023-03-31

**Authors:** M. Michalska, V. Matějka, J. Pavlovský, P. Praus, M. Ritz, J. Serenčíšová, L. Gembalová, M. Kormunda, K. Foniok, M. Reli, G. Simha Martynková

**Affiliations:** 1 Department of Chemistry and Physico-Chemical Processes, Faculty of Materials Science and Technology, VŠB-Technical University of Ostrava, 17. listopadu 2172/15, 708 00 Ostrava-Poruba, Czech Republic; 2 Institute of Environmental Technology, CEET, VŠB-Technical University of Ostrava, 17. listopadu 2172/15, 708 00 Ostrava-Poruba, Czech Republic; 3 Energy Research Centre, CEET, VŠB-Technical University of Ostrava, 17. listopadu 2172/15, 708 00 Ostrava-Poruba, Czech Republic; 4 Department of Physics, Faculty of Electrical Engineering and Computer Science, VŠB-Technical University of Ostrava, 708 00 Ostrava, Czech Republic; 5 Faculty of Science, J. E. Purkyně University, Pasteurova 15, 400 96 Usti nad Labem, Czech Republic; 6 Nanotechnology Centre, CEET, VŠB-Technical University of Ostrava, 17. listopadu 2172/15, 708 00 Ostrava-Poruba, Czech Republic

**Keywords:** Materials for energy and catalysis, Chemistry

## Abstract

Here, the comparison of two different semiconductor materials is demonstrated, TiO_2_ and melem/g-C_3_N_4_ composites—modified with balls of approximately 5 nm Ag nanoparticles (NPs) as photocatalysts for the degradation of the model dye acid orange 7 (AO7). The melem molecule synthesized here is one of a series of organic compounds consisting of triazine ring compounds with a structure similar to that of melam and melamine. The photodegradation process of AO7 was carried out to examine all powder materials as a potential photocatalyst. Additionally, two different lamps of wavelengths 368 nm (UV light) and 420 nm (VIS light) were applied to compare the photodegradation tests. A new synthesis route for the acquisition of Ag NPs (Ag content 0.5, 1.0 and 2.5 wt%), based on a wet and low temperature method without the use of reducing reagents was proposed. The best photocatalytic performances under UV and VIS light were obtained for both, TiO_2_ and melem/g-C_3_N_4_ materials (new synthesis route) modified with a very low Ag content—0.5 wt%. The photodegradation activities using UV lamp (3 h, 368 nm irradiation) for samples with 0.5 wt% of Ag: TiO_2_ and melem/g-C_3_N_4_, in excess of 95 and 94%, respectively, were achieved. The highest photoactive materials melem/g-C_3_N_4_ with 0.5 and 1 wt% Ag revealed 98% of activity under the VIS lamp after 3 h long irradiation. Our work demonstrates a novel, environmentally acceptable, and cost-effective chemical strategy for preparation of photocatalysts suitable for degradation of organic contaminants in wastewater treatment.

## Introduction

Titanium dioxide of chemical formula TiO_2_ is one of the prominent materials that has found applications in almost all research areas^1^, such as electrode material in Li-ion^2–4^ and Na-ion batteries^2,5,6^, supercapacitors^7,8^, electrochemical solar cells^9,10^, photocatalysts^11–13^ and so on. Titanium dioxide is recognized as a chemically stable semiconductor material. It shows a wide band gap energy in the range of 3.0–3.2 eV and exhibits photocatalytic activity only when exposed to UV light (up to around 390 nm)^11–13^. Electron–hole pairs photogenerated on the TiO_2_ surface show strong reducing and oxidizing properties^11–13^. The values of the edge band potentials are suitable for the creation of reactive oxygen species, for example, the oxidation of water to a hydroxyl radical (∙OH), or the oxygen reduction to a superoxide radical anion (^·^O_2_^−^), which further increase the possibility of using this material in photocatalytic processes^11–13^. To shift the light absorption of TiO_2_ to the visible range of spectra, different strategies for TiO_2_ modification were already proposed^11–13^. One of the possible approach to overcome the drawbacks mentioned above, is to dope the pristine material with different ions (i.e., N, Fe, Ni, Al, Zn, Cu)^14–16^ or modify the surface of TiO_2_ with metal, metal oxide, or carbon species^13,17–20^.

Compared to TiO_2_, the so-called bulk graphitic carbon nitride (g-C_3_N_4_, g-CN) has a proper mid-wider band gap energy (2.7–2.8 eV) to absorb visible light efficiently^21,22^. Graphitic carbon nitride can be synthesized through the thermal polymerization of precursors such as urea^23^, thiourea^24^, cyanamide^25^, dicyandiamide^23^, and melamine^26,27^. Based on our previous studies on the synthesis of a graphitic carbon nitride material, melamine is the only precursor that yields more g-CN material than other precursors^23,27^, and thus g-CN synthesis procedure is very efficient, cost-effective, and also scalable.

One of the intermediate products during the g-CN precursors polycondensation is melem. Jurgens et al.^28^ reported that crystalline powder of single-phase melem (2,5,8-triamino-tri-s-triazine) C_6_N_7_(NH_2_)_3_ was formed in sealed glass ampules by thermal treatment of several precursors (e.g., melamine C_3_N_3_(NH_2_)_3_, dicyandiamide H_4_C_2_N_4_, ammonium dicyanamide NH_4_[N(CN)_2_], or cyanamide H_2_CN_2_, respectively) at temperatures up to 450 °C. Melem possesses superior thermodynamic stability at the higher temperature range of around 400–500 °C in which is formed^29^, and Dong et al.^30^ reported that those materials could work under UV as well as VIS light.

The wide variety of applications for the graphitic carbon nitride material is due to its nontoxicity, low cost, and ease of preparation^21,22,31,32^. However, this material has a high rate of recombination of the generated charge carriers resulting in a low photocatalytic efficiency^33^. Various strategies have been applied including the fabrication of nanomaterials^32,33^ or porous structures^32,33^, doping^23,26,27,33^, metal or non-metal deposition^34–38^, coupling g-CN with other semiconductors^31,34,36,37,39^ to enhance visible light-induced photocatalytic activity of g-C_3_N_4_. Noble metal deposition, such as Ag nanoparticles (NPs), has received extensive attention due to its stronger electron storage capacity, lower cost, and nontoxicity^34–37^. Coupling of photocatalysts with silver nanoparticles brings the benefit of the increased excitons lifetime due to the fact the silver makes a Schottky barrier which enables to capture of the electron photoexcited from the valence band of a given semiconductor^40^. The increase of life-time of electron–hole pairs leads to the increase of the photodegradation activity of a given silver modified photocatalyst. SPR effect of silver on the photodegradation activity of the silver modified photocatalysts is other phenomenon that positively influences the photodegradation activity of such composites in visible light as reported, for example, Wang et al.^41^. Ag nanoparticles can be further stimulated to produce electrons and holes and act as an electron source. Additionally, they support active species and oxidize Ag to Ag^+^ due to the surface plasmon resonance (SPR) effect of silver^35,42^. Surface modified Ag-TiO_2_ nanoparticles have more effective electron traps than single Ag nanoparticles^43^. Other benefit of the silver coupling with the photocatalytic materials comprises the possible antibacterial performance of the resulting composite, and in this way, the functionality of the resulting silver modified photocatalyst is significantly widened^44^.

Herein, in this article, the comparison of the results of surface modification of TiO_2_ and melem/g-C_3_N_4_ materials using Ag nanoparticles (NPs) utilized as a photocatalyst for the degradation of the model acid orange 7 dye under UV or VIS irradiation, is demonstrated. A new synthesis route of obtaining ca. 5 nm-sized Ag NPs balls (content of Ag: 0.5, 1.0, and 2.5 wt%), based on a wet, simple chemical, and low-temperature method without using reducing reagents was proposed. All powders were extensively characterized using several complementary techniques. For the first time in this work, the new synthesized materials of melem/g-C_3_N_4_ were surface modified with Ag NPs and examined with AO7 as a potential photocatalyst.

## Methods

### Synthesis of melem/g-C_3_N_4_ composite

The pristine melem/g-C_3_N_4_ material was prepared by thermal treatment processing of the melamine precursor in air flow at 550 °C. The heating regime comprised the treatment of the 10 g of melamine placed in semi-closed alumina crucible with alumina lid at a rate of 3 °C/min to reach 550 °C with immediate displacement of the crucible out of the furnace when the 550 °C was reached. The resulting bright yellow powder was labelled g-CN-MM.

### Synthesis of silver modified TiO_2_ and melem/g-C_3_N_4_ composites

**Figure 1:**
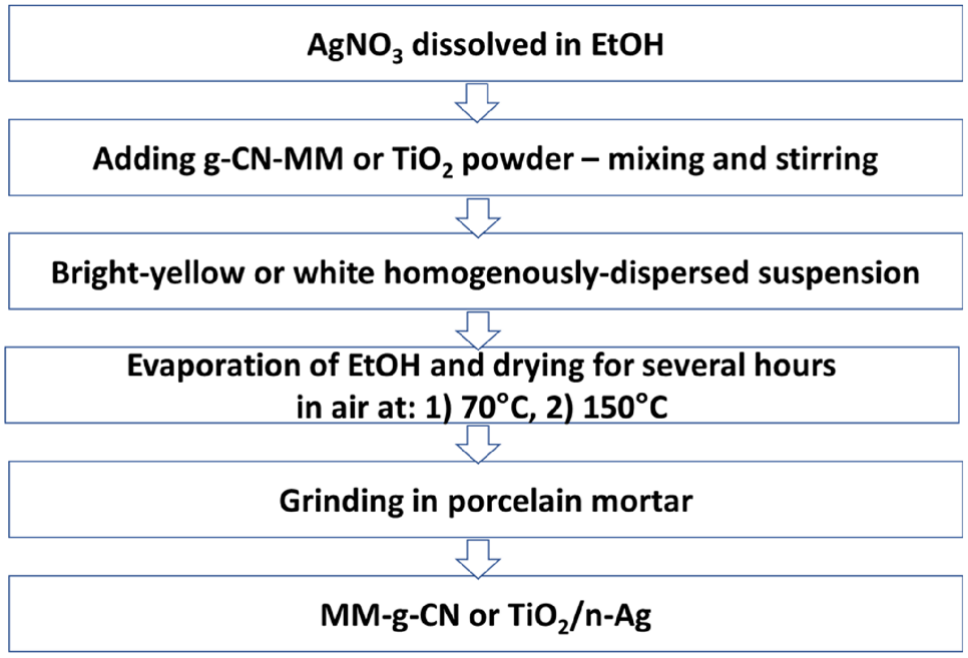
The flowchart of the synthesis of TiO_2_-A-n% Ag and of MM-g-CN-n% Ag composites.

The flowchart of the synthesis of TiO_2_ and melem/g-C_3_N_4_ decorated with Ag nanoparticles is shown in Fig. 1.

In the first step, silver nitrate (AgNO_3_, pure, Lachema) was firstly dissolved in an ethanol solution (EtOH, 96%, Merci). Then, the anatase form of TiO_2_ (Sigma-Aldrich, 99.8%) or the prepared g-CN-MM materials were added to the prepared AgNO_3_ solutions in proportions to obtain the suspensions with the Ag content of 0.5, 1.0, and 2.5 wt%, respectively. The mixtures were stirred magnetically for several hours to obtain a homogenously dispersed suspension and then dried in air for a few hours at 70 °C and then at 150 °C. The prepared samples were labelled as TiO_2_-A-*n%* Ag and MM-g-CN-*n%* Ag (where *n* indicates the amount of Ag 0.5, 1.0, 2.5 wt% in the final composites). At the last step, the composites were ground in an agate mortar to obtain a fine powder. The sample labelled as MM-g-CN was a material received after the proposed low-temperature chemical synthesis based on the treatment of g-CN-MM only in an ethanol solution (without the presence of silver).

### Characterization

Elemental analysis measurements for two samples (g-CN-MM and MM-g-CN) were performed on a CHN628 Series Carbon/Hydrogen/Nitrogen Determinator, LECO Corporation, United States. The analysis parameters were the following: furnace temperature 950 °C, afterburner temperature 850 °C, gas flow oxygen O_2_ (purity 5.0): 1. burn step was 4 L/min–10 s, 2. burn step was 0.3 L/min–180 s, 3. burn step was 1 L/min–30 s and 4. burn step was 4 L/min–30 s. The average analysis time was about 7 min. The calibration standard was used, melamine (p.a. purity, 28.58 wt% C, 4.79 wt% H, 66.63 wt% N; molecular formula C_3_H_6_N_6_) with 8 calibration points.

Powder X-ray diffraction (XRD) analysis using CuK_α_ radiation source (λ = 0.1541 nm) was employed to identify the crystalline phase of all pristine and silver-modified powders. The diffraction patterns were recorded using diffractometer Ultima IV (RIGAKU, Japan). Working conditions of all studies: CuK_α_ radiation (40 kV, 40 mA); K-beta filter; CBO selection slit—BB; Scintillation counter; continuous scan; Scan speed—4°/min; Step width—0.05°; Scan range—3–60° 2θ; Incident and receiving slit 1 – 2/3°; Receiving slit 2 – 0.6 mm were used.

Raman spectra were measured using a DXR SmartRaman dispersive Raman spectrometer (ThermoScientific, USA) with a CCD detector. The measurement parameters were as follows: excitation laser 780 nm, grating 400 lines/mm, aperture 50 μm, exposure time 1 s, number of exposures 500. An empty sample compartment was used for background measurement. Treatment of spectra: fluorescence correction (6th order).

Infrared spectra were measured by the potassium bromide pellet technique in the middle IR region. Exactly 1.0 mg of sample was ground with 200 mg dried potassium bromide. This mixture was used to prepare the potassium bromide pellets. The pellets were pressed by 8 tons for 30 s under vacuum. The infrared spectra were collected using a Nicolet iS50 FT-IR spectrometer (ThermoScientific, USA) with a DTGS detector. The following parameters were used for the measurement: spectral resolution, 4 cm^−1^; 64 scans; Happ-Genzel apodization. Treatment of spectra: polynomial (second order) baseline subtraction spectrum of pure potassium bromide.

DRS (UV–VIS) spectra of dry powder samples were measured at wavelengths ranging from 220 to 800 nm at room temperature on a Shimadzu UV-2600 Series with an IRS-2600Plus integrating sphere (Shimadzu Ltd, Japan). Powder BaSO_4_ (Nacalai Tesque, Inc., Japan) as a reference sample, and an external 2D detector was used. The values of the indirect band gap energy (E_g_) were obtained using the Tauc curve.

The photoluminescence (PL) emission spectra of the samples were measured by a FLSP920 Series spectrometer (Edinburgh Instrument Ltd, UK) in the wavelength range from 350 to 600 nm at room temperature. Spectrometer was equipped with a 450 W non-ozone xenon lamp (Steady state Xe900 lamp) and a R928P detector (PMT detector). The excitation wavelength was set at 325 nm, and excitation and emission slits 0.3 nm and dwell time 0.5 s were set for all samples of MM-g-CN group. For the samples of TiO_2_-A group, the dwell time was set at 1 s and the excitation and emission slits were set at 3 nm.

Steady-state and time-resolved photoluminescence (PL) experiments were performed using a FLS980 fluorescence spectrometer (Edinburgh Instruments, UK) equipped with a 450 W xenon arc lamp and an EPL-375 ps pulsed diode laser (λ_em_ = 372 nm with a pulse width of 66.5 ps, a repetition rate of 10 MHz and an average power of 75 µW (Edinburgh Instruments, UK) as excitation sources. PL decay curves were fitted with a multi-exponential function

1 $$I\left( t \right) = \mathop \sum \limits_{i = 1}^{3} B_{i} e^{{ - t/\tau_{i} }}$$

where *I*(*t*) is the intensity of photoluminescence, *t* is the time, *B*_*i*_ coefficients are the time-invariant constants, and *τ*_*i*_ are the decay times (decay constants). The mean decay time *τ*_*m*_ was calculated as follows

2 $$\tau_{m} = \frac{{B_{1} \tau_{1}^{2} + B_{2} \tau_{2}^{2} + B_{3} \tau_{3}^{2} }}{{B_{1} \tau_{1} + B_{2} \tau_{2} + B_{3} \tau_{3} }}$$

SEM observations were performed using a FEI Quanta 650 as well as 450 FEG (FEI, USA) scanning electron microscope equipped with an X-ray energy dispersive spectroscopy system (EDS) for chemical analysis and elemental mapping. Two different conditions of measurements as follows: Large field Low vacuum SED (LFD) Detector, Electron Beam resolution operating at HV 10 kV with Vacuum system of pressure 50 Pa (LoVac) for series of TiO_2_-A-*n%* Ag, and Everhardt Thronley SED (EDT) Detector, Electron Beam resolution operating at HV 20 kV with Vacuum system of pressure 10^−4^–10^−3^ Pa (HiVac) for series of MM-g-CN-*n%* Ag were applied. The images were collected at two different magnifications at 50 × and 40 × by using Cr layer for a series of TiO_2_-A and MM-g-CN composites with Ag, respectively.

Additionally, to compare all samples, the SEM images at 5×magnification were investigated and presented in Supplementary Material. The TEM examinations were investigated using JEOL 2100 transmission electron microscopy apparatus (Jeol Ltd., Tokyo, Japan) with an LaB6 electron gun, operating at 200 kV. The TEM images were collected by a Tengra camera (EMSIS GmbH, Münster, Germany). The powders were dispersed in an ethanol solution and, then sonicated for 5 min. One drop of the homogenously prepared dispersion was coated on a copper grid with a holey carbon film and dried at ambient temperature.

X-ray photoelectron spectroscopy (XPS) was used for characterization of the surface chemical composition of selected samples and to reveal the forms of given elements. Main parts of the XPS analyser include the high vacuum chamber which was equipped with SPECS X-Ray XR50 (Al cathode 1486.6 eV was used), hemispherical analyser SPECS PHOIBOS 100 with 5-channels detector SPECS MCD was used for the signal detection. The Flood gun SPECS FG22/35 was not used. The registered spectra were evaluated in CasaXPS software.

Photocurrent measurements were performed using a photoelectric spectrometer (Instytut Fotonowy, Krakow, Poland) and a three-electrode configuration, with Ag/AgCl and platinum wire as the reference and counter electrode, respectively. A thin layer of the material (the working electrode) was deposited at the surface of an ITO-coated transparent PET foil (60 Ω/sq resistance, Sigma-Aldrich). The sample (20 mg) was finely ground in the agate mortar with a 150 mL of ethanol. A thin layer was made out of the suspension using Elcometer Micrometric film applicator (Elcometer 3570/1, UK). The deposited uniform film was then dried at 80 °C in a dryer. The electrolyte (0.1 mol/L KNO_3_, pH = 6.1) was purged with argon for 15 min prior to and during the measurement. Photocurrents were recorded by irradiating the working electrode from the backside with a xenon lamp in the range of 250 − 450 nm with 10 nm step, applying voltages in the range between − 0.2 and 1.0 V (vs Ag/AgCl). The size of the working electrode is determined by the diameter of the window (1.0 cm), so the area of the irradiated surface is A = 1/4π cm^2^ = 0.785 cm^2^.

### Photocatalytic activity measurements

The photocatalytic activity of all prepared samples was evaluated by degradation of acid orange 7 (AO7) dye. Photodegradation of AO7 (sodium 4-[(2E)-2-(2-oxonaphthalen-1-ylidene)hydrazinyl]benzenesulfonate) at concentration 7.14∙10^−4^ mol/L was carried out under UV (368 nm) as well as VIS (420 nm) light irradiation. In a typical experiment, to obtain a homogenously dispersed suspension, 50 mg of catalyst was dispersed in a glass beaker in deionized water and magnetically stirred at 300 rpm at room temperature. Then, AO7 was added into the as-prepared suspension. To achieve the adsorption and desorption equilibrium of AO7 dye on the catalyst, all mixtures were magnetically stirred at 300 rpm, at room temperature in the dark for 60 min. Then, the UV lamp (wavelength 368 nm) or the VIS lamp (wavelength 420 nm) was turned on for UV- or VIS-light irradiation, respectively. All measurement processes were conducted at room temperature with constant stirring (300 rpm). During the selected time of the experiment (under dark or under UV/VIS light), aliquots of 2 ml of the homogenously dispersed suspension were taken using the syringe. The suspensions obtained in each time interval were filtered using a 0.20 µm pore size syringe filter (CHROMAFIL GF/RC-20/25 filters, Macherey–Nagel, Germany) to separate the photocatalytic material. The acid orange 7 degradation was controlled by observing the decrease of absorbance by using a spectrometer Helios Epsilon (Thermo Spectronic, USA) at 485 nm in a 1 cm quartz glass microcuvette against distilled water. The percentage of photocatalytic degradation of AO7 (A_sample_) for all studies samples was calculated according to the equation: A_sample_ = (1 − (C_eq. AO7_/C_0 AO7_))·100%, where C_eq. AO7_ is the equilibrium state of the concentration of AO7, and C_0 AO7_ is the initial concentration of AO7 after the dark period.

To confirm which of the reactive species are responsible for the AO7 photodegradation, the photodegradation tests were further conducted in the presence of ethylenediaminetetraacetic acid (EDTA), 1,4-benzoquinone (1,4-BQ) and t-butanol (t-Bu) as the scavengers of the holes (h^+^), the superoxide radicals (^·^O_2_^−^) and hydroxyl radicals (^·^OH). The studies were performed under UV and VIS light irradiation for pristine and 2.5 wt% Ag catalysts.

## Results and discussion

### Elemental analysis

The elemental analysis of the C, N, and H contents was performed for as prepared g-CN-MM and after its treatment in ethanol—sample MM-g-CN. The results are presented in Table 1.

**Table 1:** Elemental analysis of C, N, H contents of g-CN-MM and MM-g-CN samples.

Material	Carbon (wt%/mol%)	Nitrogen (wt%/mol%)	Hydrogen (wt%/mol%)	Oxygen* (wt%/mol%)	C:N N:C
g-CN-MM	32.5/27.1	61.7/44.0	2.70/27.5	3.10/0.019	0.53/0.61 1.90/1.63
MM-g-CN	32.3/26.7	61.8/43.8	2.78/28.1	3.12/0.019	0.52/0.61 1.91/1.64

*The amount of oxygen was calculated to 100%.

Theoretical C/N atomic ratio in melem is 0.60 considering 10 atoms of nitrogen and 6 atoms of carbon in the melem unit (there are also 6 atoms of hydrogen). The C/N molar ratio in g-C_3_N_4_ is 0.75 and that ratio in melam is 0.54 considering 6 carbon atoms and 11 nitrogen atoms per melam unit (there are also 9 hydrogen atoms in melam unit). The obtained C/N molar ratios for g-CN-MM and MM-g-CN are close to those ratios for the ideal melem unit which supports the results of XRPD indicating the melem as the major component of both samples. Also, the molar content of hydrogen in melem unit—27.2 mol% is closely similar to the contents in g-CN-MM and MM-g-CN. The oxygen is a typical contaminant of graphitic synthesized carbon nitride^45^ and melem^46^.

It should be mentioned here that our results for elemental analysis are similar to those published by Liu et al.^47^. In Liu et al. work the metal-free melem/g-C_3_N_4_ hybrid photocatalyst was prepared by hydrothermal method from previously synthesized g-C_3_N_4_ material treated at 200 °C for 12 h in water solution and finally dried at 80 °C in air^47^. It should be pointed out here that, contrary to the Liu et al. work^47^, our g-CN-MM material was synthesized only in one simple process using thermal treatment of melamine in air atmosphere at 550 °C.

To show the difference between the melamine (2,4,6-triamino-1,3,5-triazine, C_3_H_6_N_6_) precursor and samples g-CN-MM or MM-g-CN composed of melem (2,5,8-triamino-heptazine, C_6_H_6_N_10_) results from the XRD (Fig. S1A,B), Raman and FTIR (Figs. S2A,B, S3A,B), EDS with SEM (Figs. S7A,B, S8A–G), PL spectroscopy (Fig. S4) and elemental analysis (Table 1) are shown in Supplementary materials.

### X-ray diffraction analysis

**Figure 2:**
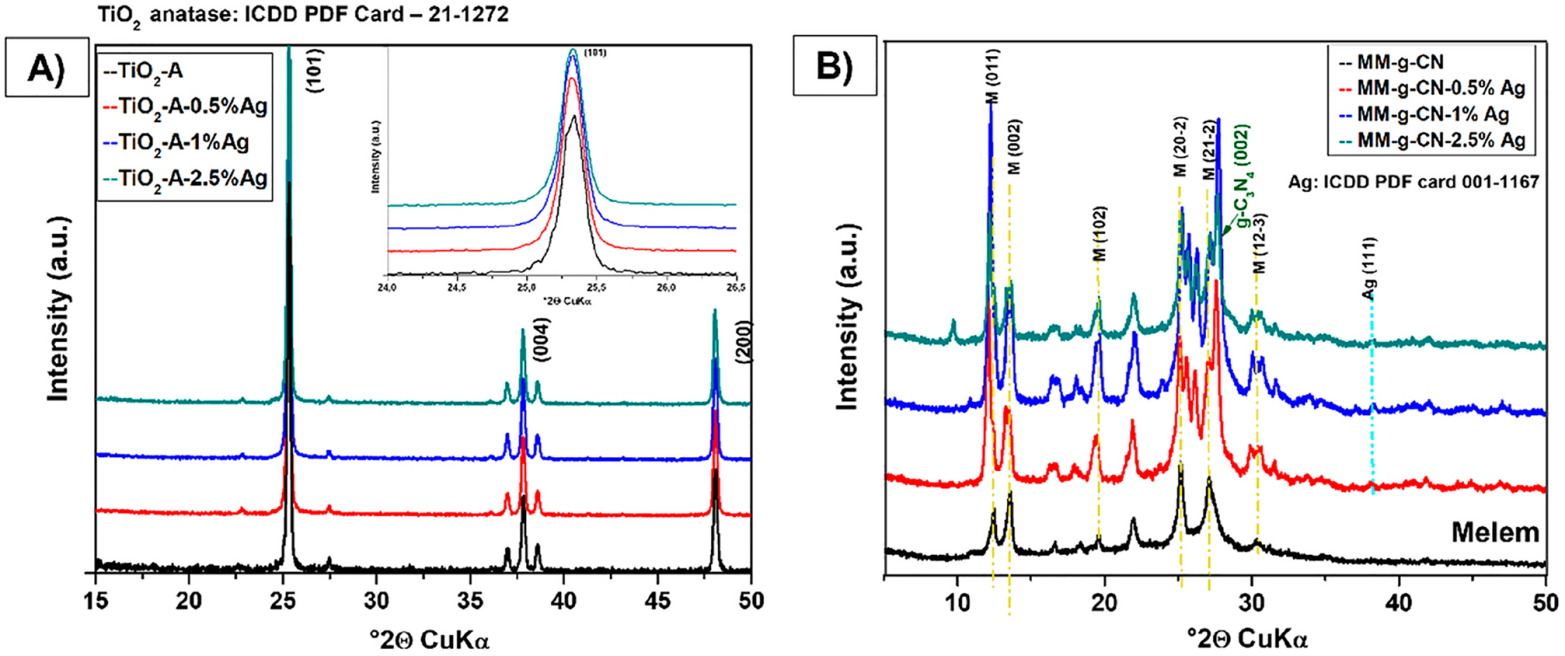
XRD patterns of: (**A**) TiO_2_ samples pure and modified with various amounts of silver. The most intensive peaks have noted lattice planes with Miller indices and detail of anatase (101) most intensive peak slight changes in position, and (**B**) melem (MM)/g-C_3_N_4_ pure and modified with 3 concentrations of Ag in composition, where the most intensive peaks of the evaluated phases are noted lattice planes with Miller indices.

To observe changes in phase and structure of studied samples, the X-ray diffraction analysis was employed. The samples were studied in as received powdered state and ambient conditions placed in a glass holder using reflection mode. The XRD analysis shows that all silver-modified TiO_2_ powders (Fig. 2A) are single-phase materials and were evaluated as tetragonal anatase phase (ICDD PDF: 21-1272) space group: I4_1_/amd. Therefore, silver decorates the surface of pristine anatase TiO_2_ and does not lead to any phase changes of TiO_2_ (Fig. 2A). The phase of silver is not detectable in any of the TiO_2_-A-n% Ag samples as a visible peak due to the low percentage of Ag in the samples and the low crystalline state compared to the major anatase phase. Observing the most intensive peaks of TiO_2_ (101)—details of Fig. 2A—we can see slight changes of peak position and intensity, however, the intensity changes are about 15% and the position of the peak maxima shifts just of hundredths of °2θ.

The XRD analysis for a series of MM-g-CN materials modified with silver nanoparticles is shown in Fig. 2B. The melem phase and g-C_3_N_4_ phase were identified based on the work of Liu et al.^48^, where diffraction data were simulated using a theoretical molecular model of melem. The process of melem/g-C_3_N_4_ phase modification with silver is causing changes that visibly broaden the major peaks and the relative intensity of peaks is increasing. Set of non-basal diffractions in the range 20–30° 2θ is exposing several new peaks compared to the pristine MM-g-CN sample. The changes are more frequent with higher concentration of Ag. The silver phase (111) is possible to detect as very small peaks of cubic pure metal visible for all modified samples.

### Raman and FTIR spectroscopy

**Figure 3:**
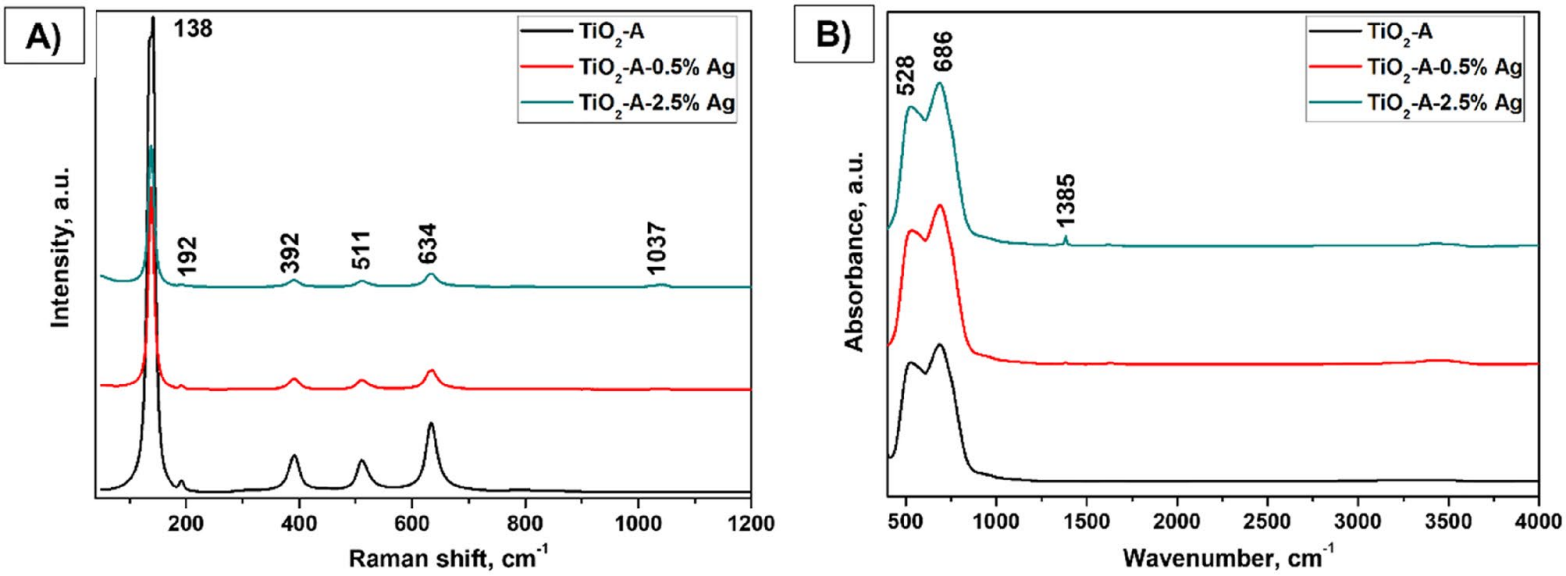
Raman (**A**) and IR (**B**) spectra of pure TiO_2_-A and TiO_2_-A-0.5% Ag and TiO_2_- A-2.5% Ag powders, where most intensive bands are noted.

To gain more information about the structural features of all materials, Raman spectroscopy analysis was used. The Raman spectra of selected samples of TiO_2_ series (TiO_2_-A; TiO_2_-A-0.5% Ag; TiO_2_-A-2.5% Ag) are shown in Fig. 3A. In the Raman spectra, the most bands belong to lattice vibration of TiO_2_ (634 cm^−1^, 511 cm^−1^, 392 cm^−1^, 192 cm^−1^, and 138 cm^−1^)^9,49^. Only the very weak band at 1037 cm^−1^ is due to a stretching vibration of nitrates as a residue after precipitation of Ag from silver nitrate. A similar effect was observed in other studies^9,50^. Further, to correlate the obtained results from Raman spectroscopy, IR studies were conducted and are shown in Fig. 3B.

**Figure 4:**
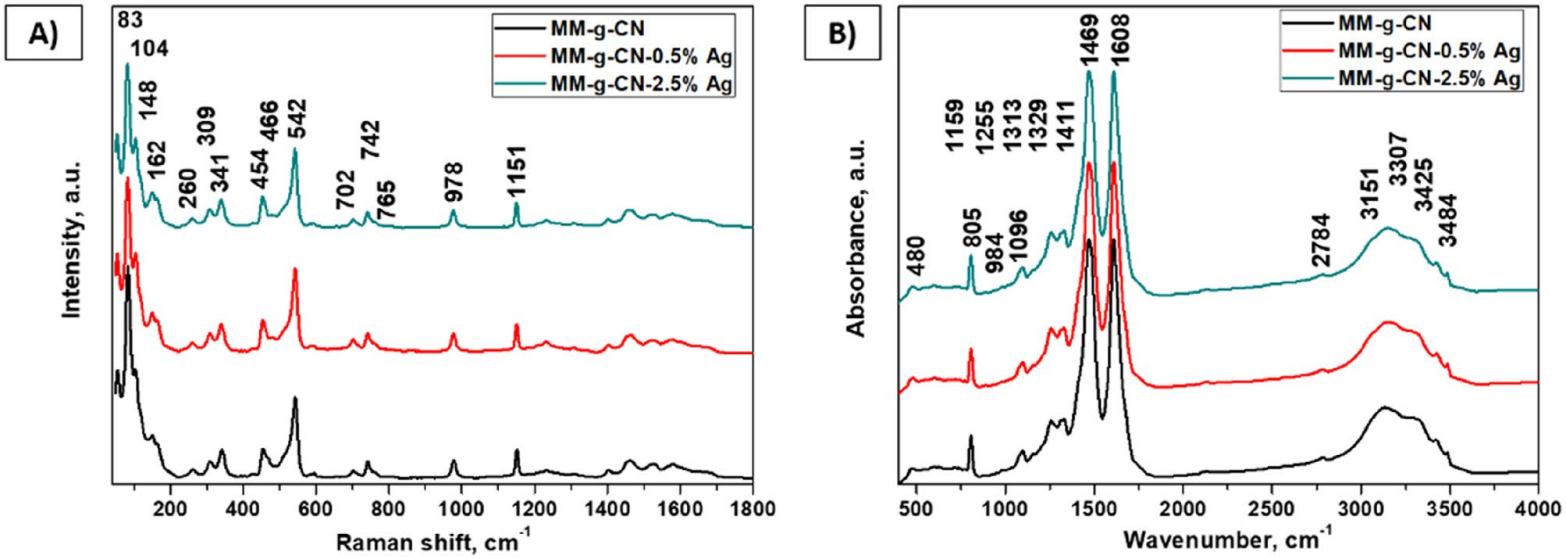
Raman (**A**) and IR (**B**) spectra of pure MM-g-CN and MM-g-CN-0.5% Ag and MM-g-CN-2.5% Ag powders, where most intensive bands are noted.

Likewise, only bands of TiO_2_ (686 cm^−1^ and 528 cm^−1^) and a very weak band of nitrates at 1385 cm^−1^ are seen in Fig. 3B. The band assignment of all the above-mentioned bands is given in Table 2. Additionally, it is clearly visible that the tetragonal I4_1_/amd structure is maintained when Ag (in the content: 0.5, and 2.5% Ag) is used to modify the surface of TiO_2_-A powders without qualitatively affecting the Raman as well as IR spectrum. Similar observations were noticed for the surface-modified MM-g-CN with Ag samples and their findings are depicted in Fig. 4.

**Table 2:** The band assignment of spectra of TiO_2_-A-n% Ag sample series.

Raman (cm^−1^)	Assignment
1037	Symmetric stretching vibration of nitrates
634	Lattice vibration of TiO_2_;
	E_g_ (doubly degenerate vibration with inverse symmetry)
511	Lattice vibration of TiO_2_;
	A_1g_ (non-degenerated vibration with inverse and mirror symmetry)
392	Lattice vibration of TiO_2_;
	B_1g_ (non-degenerated vibration, asymmetric with inverse and mirroring)
192	Lattice vibration of TiO_2_;
	E_g_ (doubly degenerate vibration with inverse symmetry)
138	Lattice vibration of TiO_2_;
	E_g_ (doubly degenerate vibration with inverse symmetry)

IR	Assignment
1385	Asymmetric stretching vibration of nitrates
686	Stretching vibration of TiO_2_
528	Stretching vibration of TiO_2_

The Raman spectra of selected samples of the MM-g-CN series (MM-g-CN; MM-g-CN-0.5% Ag; MM-g-CN-2.5% Ag) are shown in Fig. 4A. The significant bands visible in the spectra could be assigned to melem. There are mainly bands of triazine ring (1151 cm^−1^, 978 cm^−1^, and 542 cm^−1^), amino group (466 cm^−1^ and 454 cm^−1^), and the lattice vibration of melem (148 cm^−1^, 104 cm^−1^, and 83 cm^−1^)^45–47,49–53^. The IR spectra of MM-g-CN sample series are shown in Fig. 4B.

The spectra contain characteristic bands of amino group (3484 cm^−1^, 3425 cm^−1^, 3307 cm^−1^, 3151 cm^−1^, and 1608 cm^−1^) and triazine ring (1469 cm^−1^, and 805 cm^−1^)^47–54^. Especially bands at 1608 cm^−1^ and 1469 cm^−1^ are entirely characteristic for the melem spectrum^47–54^. The band assignment of all the above-mentioned bands is given in Table 3.

**Table 3:** The band assignment of spectra of both MM-g-CN-n% Ag sample series.

Raman	Assignment
1151	Stretching vibration of triazine ring
978 542	Deformation vibration of triazine ring (out of plane)
	Vibration of triazine ring
466 454	Combination bands of deformation (bending) vibration of -NH_2_ and deformation (rocking) vibration of -NH_2_
148	Lattice vibration of melem
104	Lattice vibration of melem
83	Lattice vibration of melem

IR	Assignment
3484	Asymmetric stretching vibration of -NH_2_
3425	Symmetric stretching vibration of -NH_2_
3307	Stretching vibration of -NH_2_
3151	Combination band of deformation vibration of -NH_2_ and stretching vibration of C-N
1608	Deformation vibration of -NH_2_
1469	Symmetric stretching (breathing) vibration of triazine ring
805	Deformation vibration of triazine ring (out of plane)

Both above-mentioned spectra are entirely typical for melem and melamine. The band assignment of both Raman and infrared spectra of melamine is given in Table S1.

### Diffuse Reflectance spectroscopy studies

**Figure 5:**
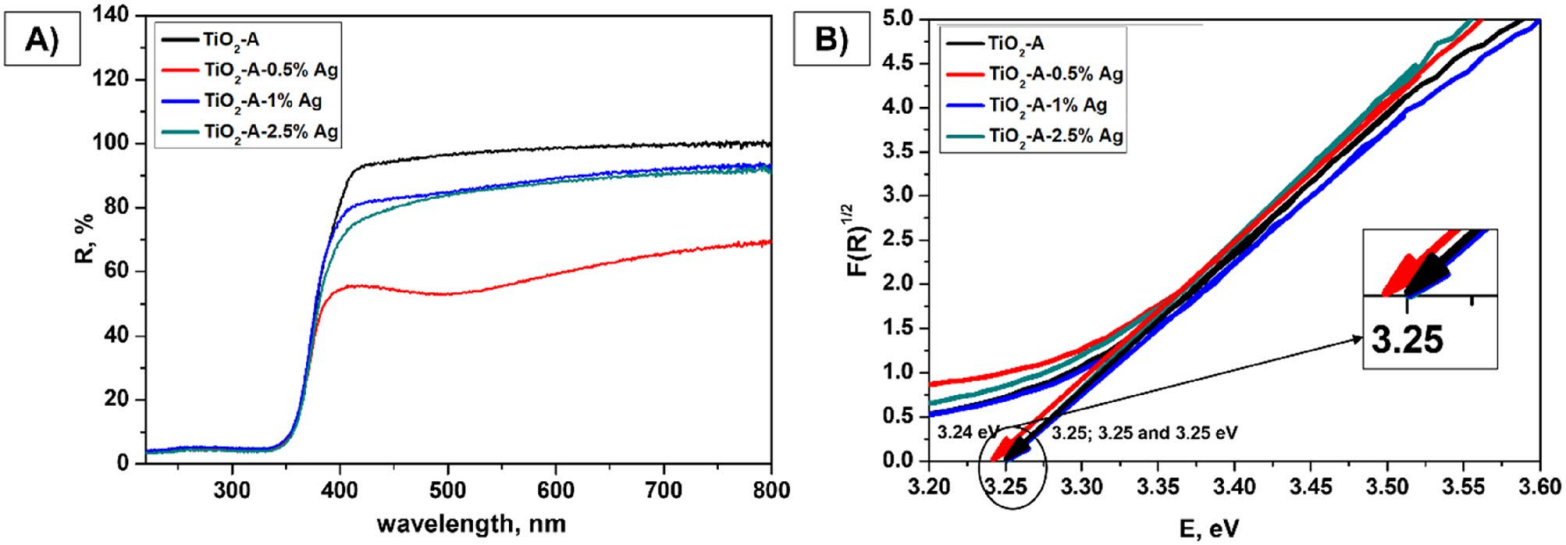
Diffuse reflectance UV–Vis spectra (**A**) and Tauc spectra (**B**) at wavelengths from 220 to 800 nm of TiO_2_-A-n% Ag.

**Figure 6:**
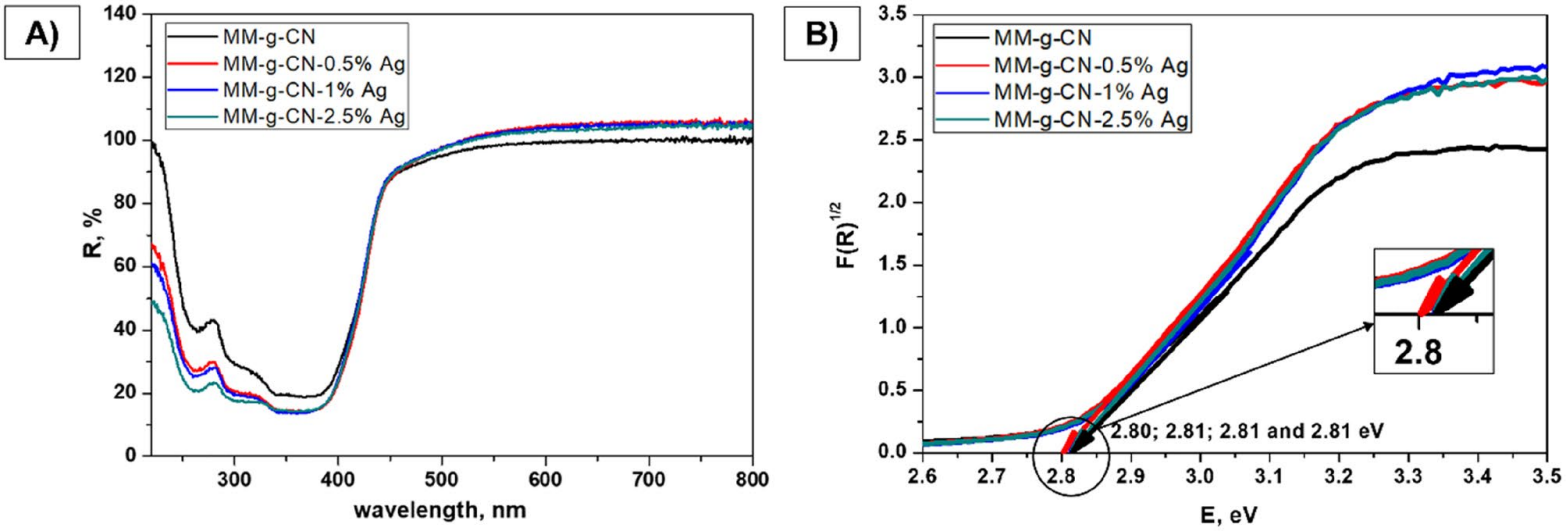
Diffuse reflectance UV–Vis spectra (**A**) and Tauc spectra (**B**) at wavelengths from 220 to 800 nm of pure MM-g-CN and MM-g-CN-n% Ag.

Diffuse Reflectance spectroscopy (DRS) analysis was used to determine the effect of modification of two selected semiconductor materials with silver nanoparticles within the registered spectra in the UV–VIS region 220–800 nm. That quick, cheap, and non-destructive technique allows getting the values of band gap energy and to observe the effect of an increasing concentration of Ag on the surface of pristine semiconductor materials. Later, the achieved results are correlated with photoluminescence studies (see chapter 3.5). The DRS spectra and Tauc curves from which the E_g_ values were determined for the series of TiO_2_-A and MM-g-CN powders are shown in Figs. 5(A,B), and 6(A,B).

The indirect band gap energy values (Tauc spectra obtained from Kubelka–Munk function) evaluated from DRS spectra of TiO_2_-A and MM-g-CN, both with and without Ag are listed in Tables 4 and 5.

**Table 4:** Indirect band gap energy values (Kubelka–Munk function, Tauc spectra) from DRS spectra of TiO_2_-A-n% Ag.

Materials	E_g_ (eV)
TiO_2_-A	3.25
TiO_2_-A-0.5% Ag	3.24
TiO_2_-A-1% Ag	3.25
TiO_2_-A-2.5% Ag	3.25

**Table 5:** Indirect band gap energy values (Kubelka–Munk function, Tauc spectra) from DRS spectra of MM-g-CN-n% Ag.

Materials	E_g_ (eV)
MM-g-CN	2.81
MM-g-CN-0.5% Ag	2.80
MM-g-CN-1% Ag	2.81
MM-g-CN-2.5% Ag	2.81

Table 4 and 5 show that E_g_ values obtained for samples from the TiO_2_-A as well as MM-g-CN group are almost the same. Here it is worth noting that the content of Ag was not very high, so the effect of increasing Ag concentration on the surface of both materials, and thus the obtained differences of E_g_ values are negligible. The E_g_ values obtained for a series of TiO_2_-A and MM-g-CN samples are similar to the values presented by authors^48,55,56^.

The optical properties of Ag-based TiO_2_-A-n% materials are shown in Fig. 5A. A very sharp absorption edge for pure TiO_2_-A was observed at about 400 nm (E_g_ around 3.10 eV) as an intrinsic absorption of TiO_2_^55,56^ and the band gap energy 3.25 eV was then estimated from the Tauc plot (Fig. 5B). For the silver modified TiO_2_-A samples, the reflectance increases with growing content of Ag which indicates, the samples absorb more light in the Vis region. Silver-modified TiO_2_-A-n% Ag samples show a wider band in the DRS spectrum (Fig. 5A) with a wide absorption centred at around 510 nm (especially visible for TiO_2_-A-0.5% Ag), the presence of this phenomenon indicates the localized surface plasmon resonances (LSPR) effect caused by the presence of silver nanoparticles^55^. This effect is significant especially for the material TiO_2_-A-0.5% Ag.

For the samples of MM-g-CN series, the DRS spectra shown in Fig. 6A indicate that all materials have an absorption at about 440 nm (2.81 eV) (Table 5), which corresponds well with the band gap energy values of this type of material presented in the literature^36,48^. Silver-modified MM-g-CN samples show a significant enhancement of absorption in the visible light regions from 400 to 800 nm, which is attributed to the SPR (Surface Plasmon Resonance) effect of Ag nanoparticles^56^.

### Photoluminescence studies

**Figure 7:**
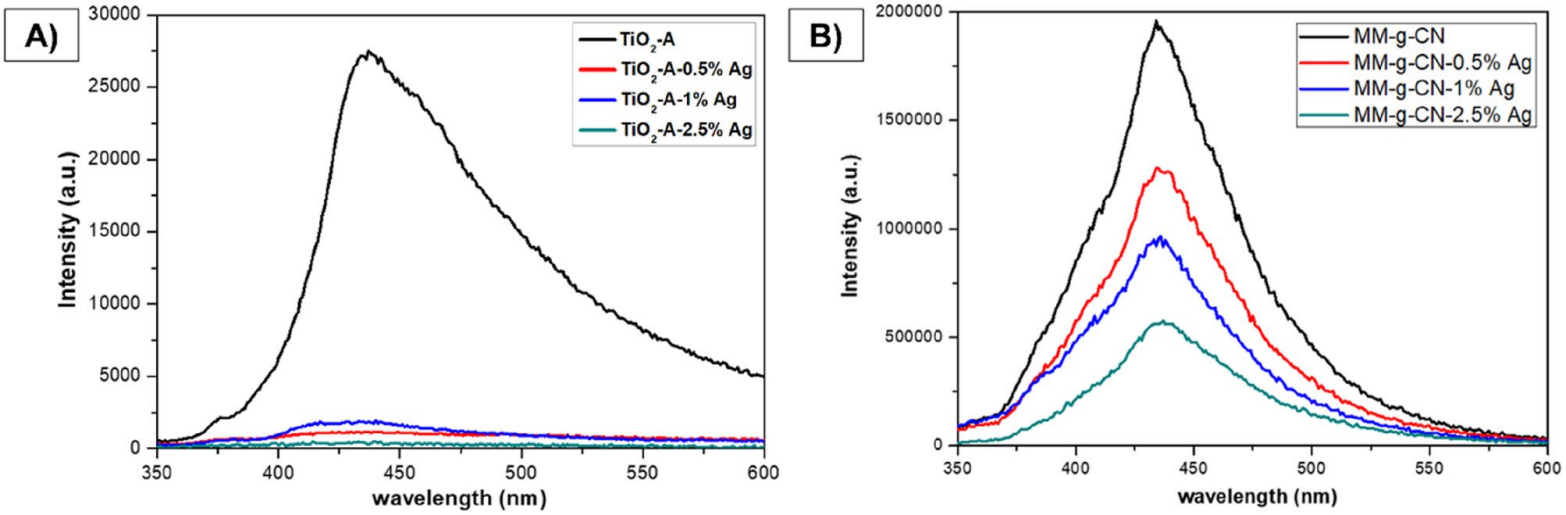
PL spectra of: (**A**) TiO_2_-A-n% Ag and (**B**) MM-g-CN-n% Ag powders.

Photoluminescence (PL) spectra of TiO_2_-A and TiO_2_-A-n% Ag, and MM-g-CN and MM-g-CN-n% Ag samples are shown in Fig. 7.

TiO_2_-A sample showed the highest intensity of the maximum of PL peak, but with increasing Ag content the intensity of this peak decreased (Fig. 7A). The maximum of PL intensity is blue-shifted up to 1 wt% of Ag in comparison to TiO_2_-A, while the further increase of Ag to 2.5 wt% caused the red shift in comparison to the sample with 1 wt% of Ag, but still blue-shifted in comparison to sample TiO_2_-A, see Table 6.

**Table 6:** Maximum emission bands of pristine TiO_2_-A and modified with Ag NPs.

Materials	Maximum emission bands (nm)
TiO_2_-A	437
TiO_2_-A-0.5% Ag	432
TiO_2_-A-1% Ag	428
TiO_2_-A-2.5% Ag	433

The intensity of the PL signal of pure MM-g-CN is very strong, and when the Ag content in the samples increases, the PL intensity subsequently decreases (Fig. 7B), while the PL maxima are weakly red-shifted (Table 7) from 435 to 438 nm.

**Table 7:** Maximum emission bands of pristine MM-g-CN and modified with Ag NPs.

Materials	Maximum emission bands (nm)
MM-g-CN	434
MM-g-CN-0.5% Ag	435
MM-g-CN-1% Ag	436
MM-g-CN-2.5% Ag	438

The results obtained from photoluminescence measurement for MM-g-CN samples are aligned with the literature^34,35,48^. In the Supplementary Material, the deconvolution of the PL spectrum for sample MM-g-CN is presented (Fig. S4).

The deconvolution of the PL spectra of MM-g-CN to the three individual components shown in Fig. S4 was performed using the Gaussian function in the OriginPro 8 software. It can be seen that the MM-g-CN sample has a weak peak centred at 396 nm, which corresponds to melam, and a peak centred at 435 nm, which corresponds to melem.The band at 452 nm corresponds to g-C_3_N_4_. The obtained results perfectly matched to the work published by Liu et al.^48^. The authors showed, that melem and g-C_3_N_4_ with maxima centred around 435 and 452 nm are the main contributors to the registered PL band, and the presence of melam is reflected by the presence of weak PL peak with the maxima centred at 396 nm^48^. It should be pointed out here, that the materials studied by Liu et al.^48^ were synthesized under different conditions in comparison to our samples as already mentioned.

In general, the high intensity of the PL spectrum of g-C_3_N_4_ reflects fast electron–hole recombination, usually resulting in lower photoactivity^44^. Defects in the g-C_3_N_4_ structure cause rapid recombination of the electrons with photoexcited holes^37^. The information derived from PL spectra supports also the experiments with photodegradation of AO7 as further shown and discussed in subchapter 3.9.

For photogenerated electrons in g-C_3_N_4_ bulk material, the conduction band (CB) has a much greater power for reduction reactions compared to TiO_2_^57^. The Ag nanoparticles found on the surface of the g-C_3_N_4_ bulk material could abate the charge recombination acting as an electron sink, efficiently trapping photogenerated electrons from the conduction band of g-C_3_N_4_ (over the band gap energies of 2.83 eV for the MM-g-CN-2.5% Ag material can be explained by the recombination process of electrons and holes)^22^.

### PL decay study

**Figure 8:**
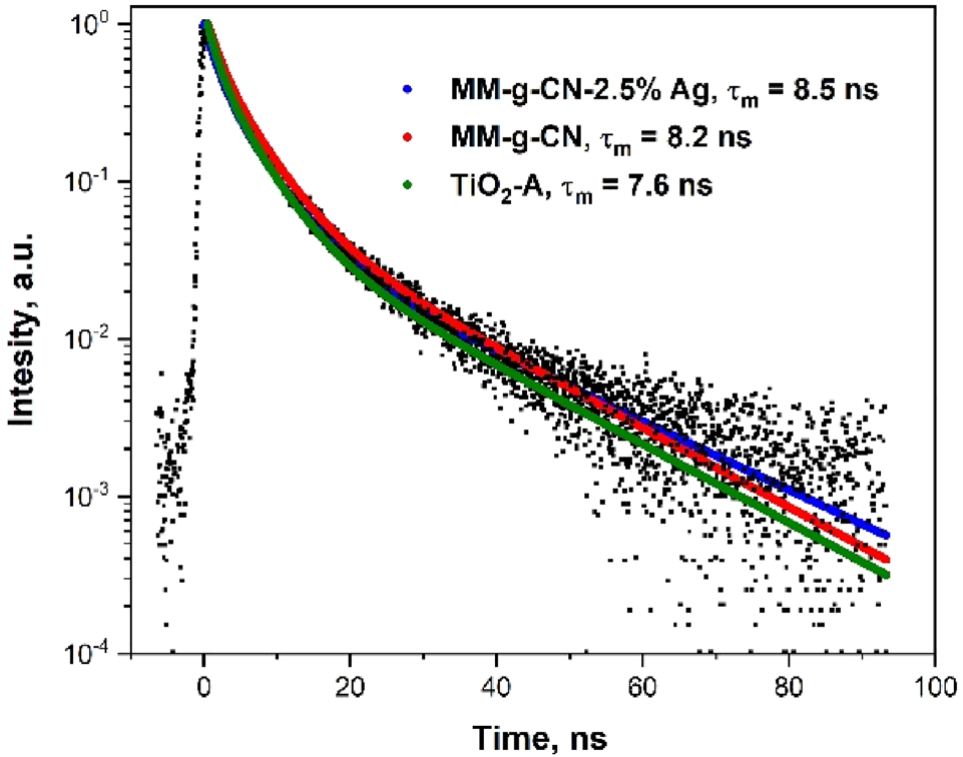
Normalized PL decay curves of MM-g-CN-2.5% Ag, MM-g-CN, and TiO_2_-A.

PL decay curves were recorded for us to understand photocatalytic processes in the studied materials. The lifetimes of photoinduced electrons and holes were calculated by fitting the PL decay curves with selected exponential relationships and by calculation of mean decay times according to Eqs. (1) and (2), respectively. The normalized PL excitation and emission spectra (Figs. S5, S6) of the materials showed broad excitation bands around 300 nm and emission bands around 440 nm and, therefore, a laser operated at the wavelength of 372 nm could be used for the excitation of the studied samples except TiO_2_-A-2.5% Ag. The best fits of the normalized PL decay curves displayed in Fig. 8 were obtained for the 3rd order kinetic model as documented by Chi square (Χ^2^) values given in Table S2 (Supplementary materials). The calculated mean decay times τ_m_ are given in Fig. 8 as well.

The mean decay times of electrons and holes photoinduced in MM-g-CN and MM-g-CN-2.5% Ag were of similar values: 8.2 ns and 8.5 ns, respectively. Considering the PL spectra and the photocatalytic time plots it implies that the excited electrons in g-C_3_N_4_ were partially trapped in the Ag nanoparticles and, therefore, they did not recombine with holes providing the PL irradiation. In turn, the electrons were involved in the photocatalytic degradation of AO7. Unlike the photocatalytic experiments performed under UV irradiation, using VIS irradiation (420 nm) the surface plasmon resonance (SPR) effect was initiated in the Ag nanoparticles. Magnetic field induced by the SPR facilitated the formation of electrons and holes in g-C_3_N_4_^58^. Moreover, hot electrons^68^ with higher energy from the Ag SPR excitation were likely transferred from the Ag NPs to the conduction band of g-C_3_N_4_ enhancing its photocatalytic activity^58–60^.

The shorter mean decay times of 7.6 ns of TiO_2_-A documents the faster recombination of electrons and holes but we cannot compare it with that of TiO_2_-A-2.5% Ag. However, the above-described process based on the transfer of hot electrons from the Ag NPs to the conduction band of TiO_2_ explains the observed photocatalytic activity of TiO_2_-A-2.5% Ag under VIS irradiation, see Fig. 14C.

### Scanning and transmission electron microscopy examination

**Figure 9:**
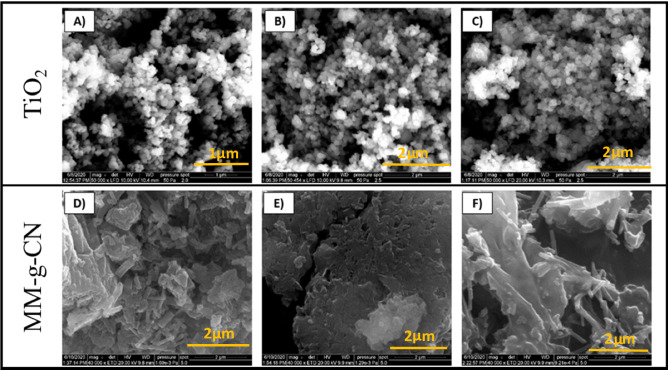
SEM images of: (**A**) TiO_2_-A, (**B**) TiO_2_-A-0.5% Ag, (**C**) TiO_2_-A-2.5% Ag at 50,000×magnification, and (**D**) MM-g-CN, (**E)** MM-g-CN-0.5% Ag, (**F**) MM-g-CN-2.5% Ag at 40,000×magnification.

To support the structural and spectroscopy features and get more information about our materials as well as before their testing for photocatalysis, the morphology examinations (SEM with EDS, TEM) were performed. Further, these pivotal analyses had other tasks, such as visualizing changes in morphology of the pristine materials after the proposed silver modification easy, straightforward and cost-effective approach. The quintessence of these measurements was also the confirmation of silver nanoparticles on the surface of both semiconductor materials. The scanning electron microscopy (SEM) images of TiO_2_-A and MM-g-CN materials pristine and modified with silver nanoparticles are presented in Fig. 9.

It can be seen that the pristine titanium dioxide (Fig. 9A) material and modified with 0.5 and 2.5 wt% of Ag (Fig. 9(B, C)) are homogenous and represent the globular structure. Hence, the silver nanoparticles on SEM micrograph (see Fig. 9) as well as on EDS mapping of the selected elements (both measurements were performed for the sample with 2.5 wt% of Ag, see Fig. S5A in Supplementary Material) appear as small balls.

**Figure 10:**
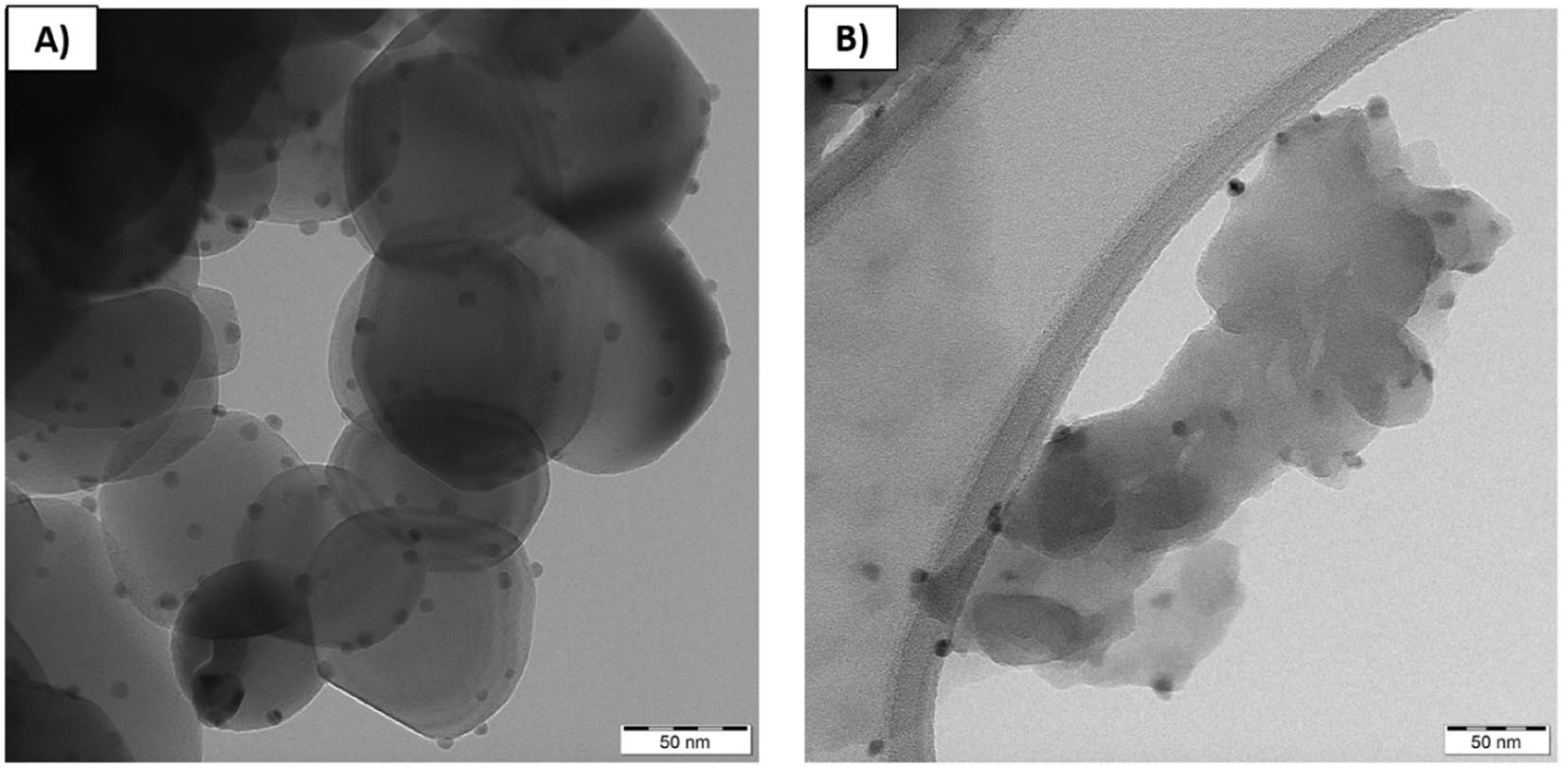
TEM pictures of: (**A**) TiO_2_-2.5% Ag and (**B**) MM-g-CN-2.5% Ag powders.

The morphology that depicts the series of MM-g-CN with Ag was different from the series with TiO_2_-A. The layered structure with belts is observed only for the pristine material—sample MM-g-CN (Fig. 9D). The MM-g-CN composites with Ag (Fig. 9(E, F)) are strongly agglomerated in comparison to the pristine material. One can observe that after introduction of silver ions into the g-CN-MM-EtOH solution and drying the materials (see scheme in Fig. 1), the destruction of layered sheets created a dendrite of about 1 μm in length (Fig. 9F). We also presume that the Ag balls cannot be seen because of the presence of Cr layer which was sputtered over the samples prior to the SEM observation to suppress the charging of the samples from MM-g-CN group during the measurement. To confirm the presence of Ag in our samples, the EDAX analysis was performed. The studied two samples TiO_2_-A-2.5% Ag and MM-g-CN-2.5% Ag revealed: 1.95 and 1.85 wt% of Ag, respectively. To check the presence of Ag nanoparticles in the MM-g-CN-2.5% Ag sample the EDS mapping was collected (see Fig. S5B in Supplementary Material), and the similar observation to TiO_2_-A-2.5% Ag material was visible. The Ag NPs existed as small balls. TEM images collected for TiO_2_-A-2.5% Ag and MM-g-CN-2.5% Ag powders show the homogenous distribution of Ag nanoparticles (see Fig. 10(A,B)).

The TEM images depicted approximately 5 nm-sized silver nanoparticles balls on the surface of both analyzed samples, respectively. The TEM results for the TiO_2_-A-2.5% Ag sample were in accordance with SEM examinations. These results confirm the effectiveness of the developed method of modifying the surface of both semiconductor materials with Ag NPs without the presence of reducing agent. In Supplementary Material, the SEM images of all materials: melamine, g-CN-MM, MM-g-CN, and TiO_2_-A with Ag are summarized in Fig. S8A–G at 5000×magnification. The well-crystalline structure shows only melamine, as is seen in Fig. S8A.

### XPS spectroscopy

To further investigate the surface chemical composition of the materials, XPS measurements were performed. The surface elemental compositions of the samples are listed in Table 8.

**Table 8:** The elemental composition of the samples revealed by XPS measurement.

Sample	Ag (at%)	C (at%)	O (at%)	Ti (at%)	O/Ti
TiO_2_-A		14.9	60.4	24.7	2.4
TiO_2_-A-2.5% Ag	5.8	19.9	54.6	19.7	2.8

Sample	Ag (at%)	C (at%)	N (at%)	O (at%)	N/C
MM-g-CN		44.6	53.7	1.7	1.2
MM-g-CN-2.5% Ag	1.6	39.5	55.6	3.4	1.4

The TiO_2_-A sample is composed of Ti, O and C, as supposed the sample TiO_2_-A-2.5% Ag in addition consists of Ag. Table 8 shows that the O/Ti ratio for both samples is higher than 2, and it is most probably caused by the presence of OH groups on the surface, the presence of OH groups is also confirmed by the weak bands in FTIR spectra (see Fig. 3B). The high content of carbon is caused by the combination of the contamination and contribution of carbon adhesive tape.

**Figure 11:**
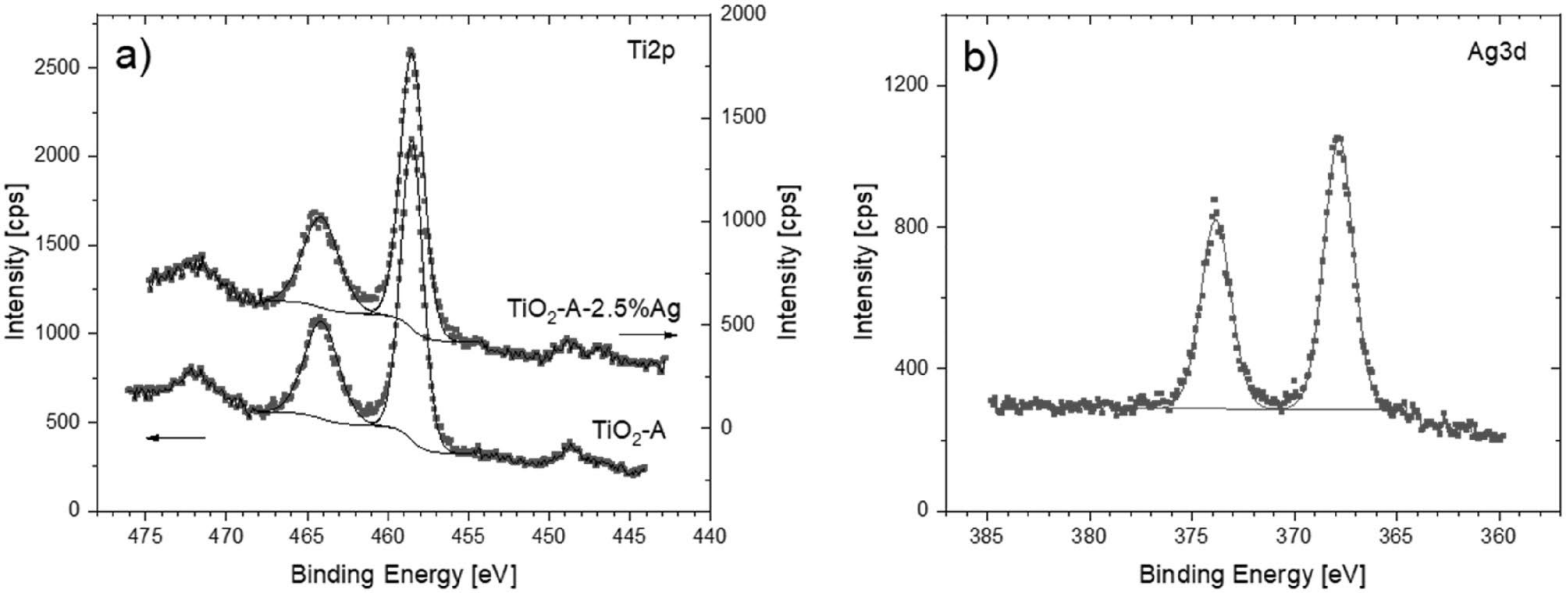
High resolution XPS spectra of Ti2p (**a**) and Ag3d (**b**).

The collected high resolution Ti2p spectra (Fig. 11a) are typical for TiO_2_ materials with one doublet at bonding energies at 458.5 eV and 454.1 eV for Ti2p3/2 and Ti2p1/2, respectively^61^.

Because the changes are not observed in Ti2p spectra (Fig. 11a) registered for the sample TiO_2_-A-2.5% Ag if compared to those spectra registered for the sample TiO_2_-A, it supports that Ag forms nanoparticles on top of TiO_2_ and Ag is not incorporated into TiO_2_^62^.The silver detected in samples TiO_2_-A-2.5% Ag has a single doublet character with relatively low FWHM at binding energy 367.8 eV and 373.8 eV for Ag3d5/2 and Ag3d3/2 (Fig. 11b). The spin split between both spin states is exactly expected 6 eV which is typical for all states of silver. Silver is known for to be difficult to differentiate the state by XPS^62^, the other techniques, for example electron energy loss spectroscopy (EELS) could be used for confirmation of the silver states too. Narrow Ag3d peaks at low binding energy could indicate the presence of metallic Ag in TiO_2_-A-2.5% Ag sample, on the other hand the Auger parameter between Ag MNN and Ag3d5/2 (716.93 eV) of this sample indicates the presence of Ag_2_O.

**Figure 12:**
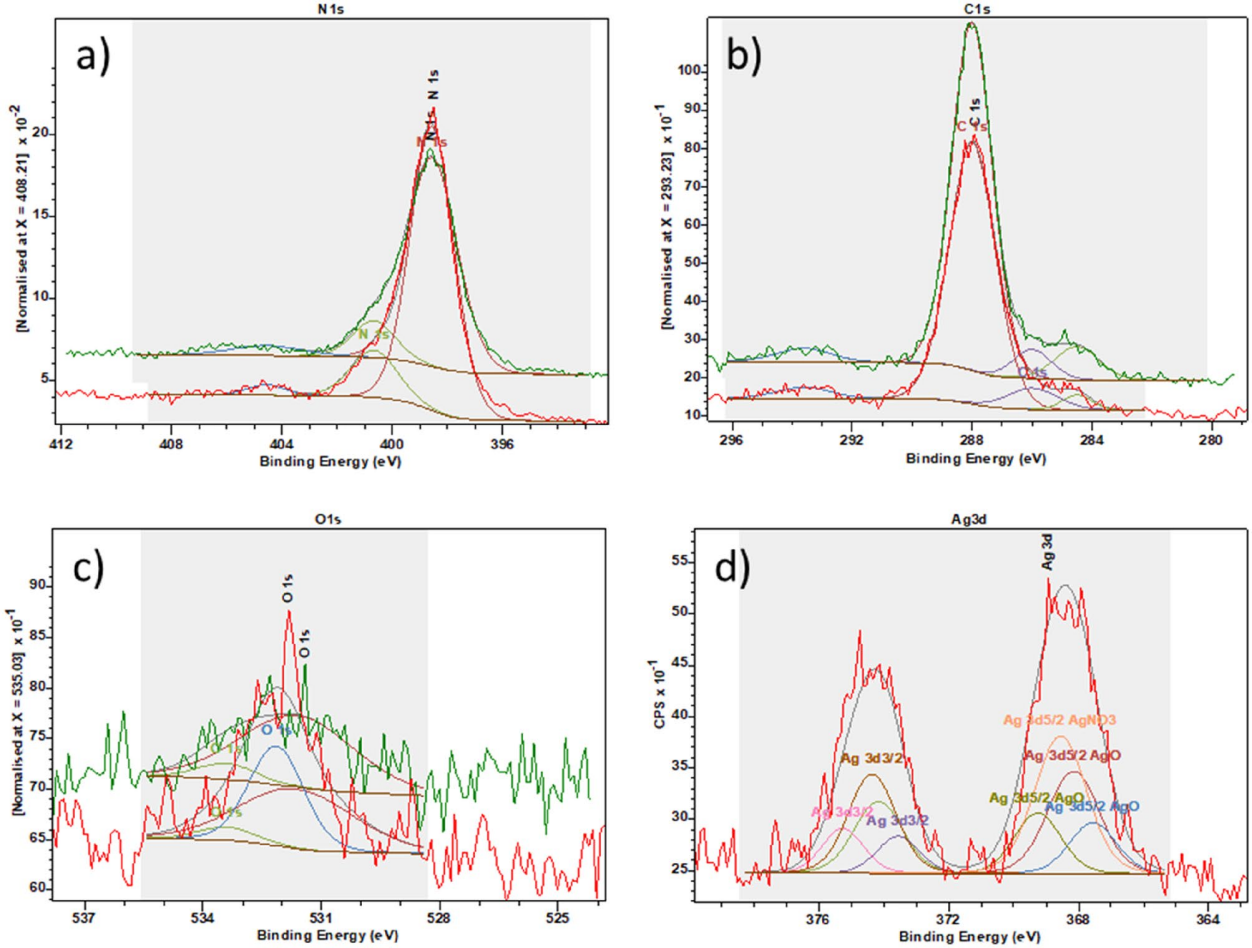
High resolution XPS spectra of N1s (**a**), C1s (**b**), O1s (**c**) and Ag3d (**d**) registered for the samples MM-g-CN (green) and MM-g-CN-2.5% Ag (red).

High resolution XPS spectra including N1s, C1s and O1s peaks registered for samples MM-g-CN and MM-g-CN-2.5% Ag and Ag3d peaks registered for MM-g-CN-2.5% Ag are shown in Fig. 12.

The N1s spectra of both samples (Fig. 12a) consists of three main components at 400.6 eV, 398.5 eV and 404.6 eV. The first two peaks correspond to both types of sp^2^ hybridized nitrogen—Nα bonded to two neighbouring carbon atoms (C–N=C) and Nβ a tertiary nitrogen N–(C)_3_ group, respectively. The third weaker component can be attributed to –NH_2_ or = NH groups^27^.

The C1s spectrum (Fig. 12b) can be deconvoluted into three peaks at 284.5 eV, attributable to C–C/C–H bonds, 286.3 eV indicating a probable small contribution of C–O bonded carbon, but more probably a C–N component, and the third component at 288 eV is identified as sp2 bonded carbon in C=N group ^63^.

Broad and asymmetric O1s peak of measured samples (Fig. 12c) was deconvoluted into two components. The first one, at higher binding energy (533.0 eV) is associated to the surface adsorbed oxygen or water^61^. The second one centred at 531.2 eV, was interpreted in accordance with literature as oxygen incorporated in g-C_3_N_4_ structure^27^.

The presence of silver in the sample MM-g-CN-2.5% Ag has practically no influence on the XPS spectra of C1s and N1s (compare the spectra in Fig. 12(a,b), but the addition of Ag increases oxygen content, as evident in Table 8. The Ag3d spectrum of MM-g-CN-2.5% Ag sample (Fig. 12d) is different from Ag observed in TiO_2_-A-2.5% Ag (Fig. 11b). The shape of Ag MNN and Ag3d indicate a more complex structure and the best agreement was found with a mixture of AgO and AgNO_3_^62^. Although the results of XPS did not directly prove the presence of metallic silver in sample MM-g-CN-2.5% Ag, its presence cannot be excluded and further investigation using already mentioned EELS method could be performed to reveal the presence of metallic silver.

### Electrochemical studies

**Figure 13:**
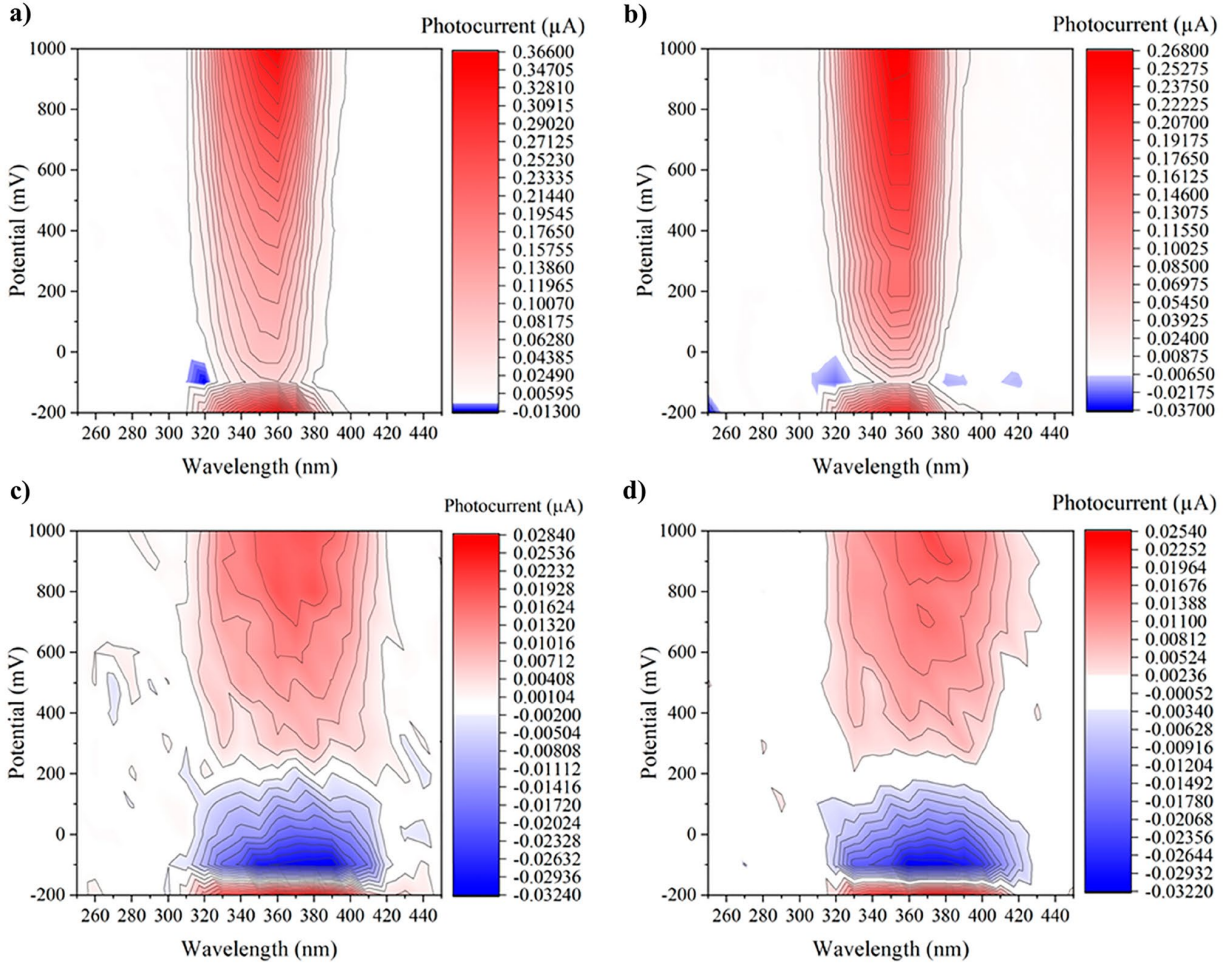
Photocurrent generation as a function of potential and wavelength for (**a**) TiO_2_-A; (**b**) TiO_2_-A-0.5% Ag, (**c**) MM-g-CN, and (**d**) MM-g-CN-0.5% Ag.

Electrochemical studies were performed for the unmodified samples TiO_2_-A and MM-g-CN, as well as for these samples modified with 0.5 wt%. All of the selected samples were subjected to the evaluation of their ability for the generation of photocurrent. The photocurrent data recorded as function of wavelength and applied potential are reported in Fig. 13. The blue areas represent cathodic photocurrent (corresponding to the reduction of an electron acceptor) and the red areas represent anodic photocurrent (corresponding to oxidation of an electron donor, mainly water in aqueous electrolyte).

Based on the photocurrent generation measurements it is clear that samples containing CN show higher reduction “strength” at lower potentials (see Fig. 13A,B). In addition, samples with CN showed photocurrent generation at higher wavelengths (up to 420 nm). In comparison, TiO_2_-A samples showed no photocurrent above 390 nm (Fig. 13C,D).

### Degradation of AO7 under UV or VIS light

The own photodegradation experiment consists of two periods: (i) dark period, and (ii) irradiation period. In the dark period, an adsorption–desorption equilibrium was reached for all of the samples, what is signalized by the negligible variation in relative change of AO7 concentration during this period (see Fig. S9 in Supplementary material).

**Figure 14:**
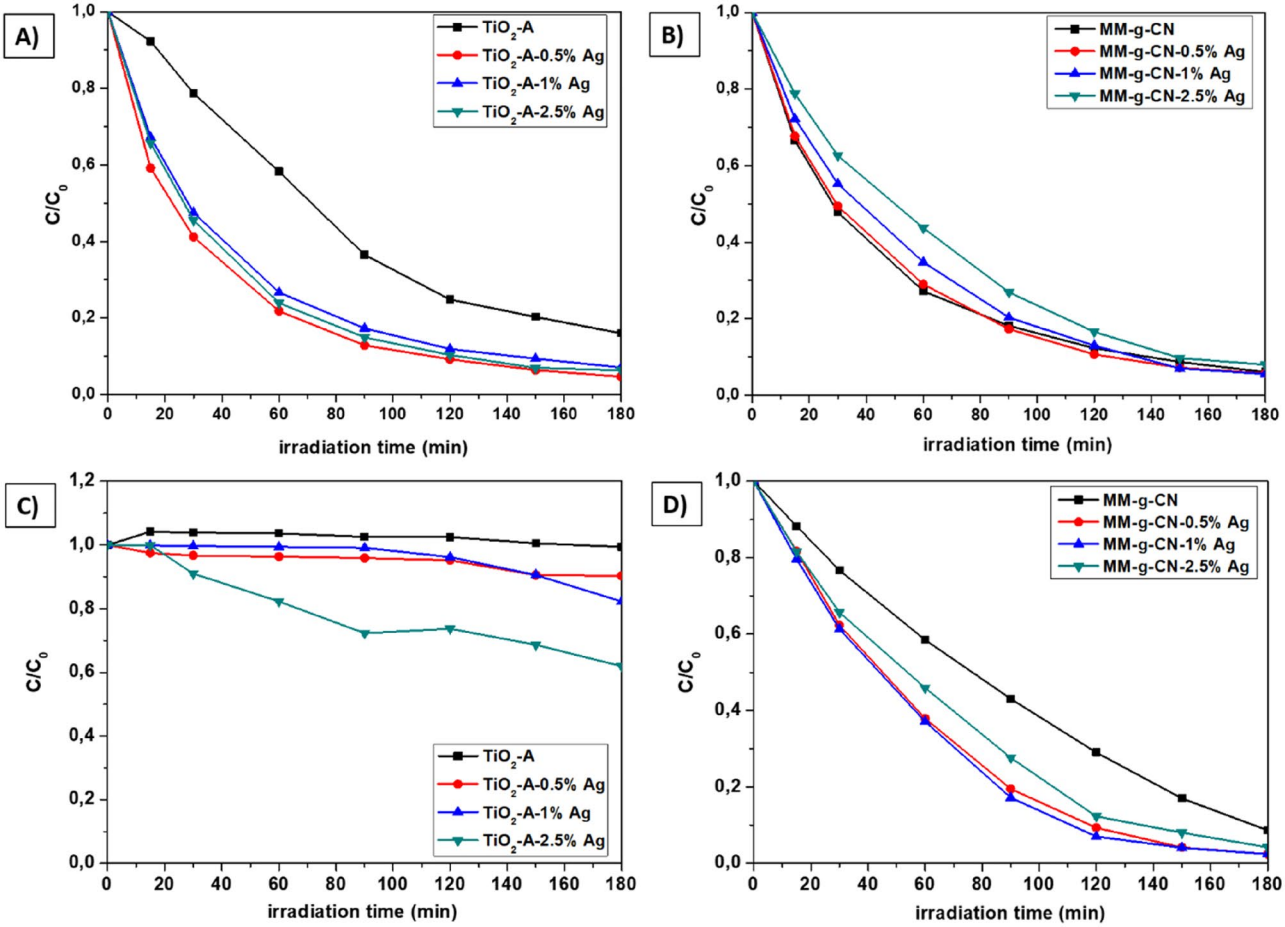
Photocatalytic degradation processes of AO7 for TiO_2_-A-n% Ag (**A**,**C**) and MM-g-CN-n% Ag (**B**,**D**) samples. (**A**,**B**)—UV lamp irradiation; (**C**,**D**)—VIS lamp irradiation. Concentration of AO7: 7.14 × 10^–4^ mol/L; mass of the catalyst: 50 mg.

The time dependency of the extent of photocatalytic degradation of AO7 with samples of TiO_2_-A and MM-g-CN series under UV or VIS irradiation (2nd period of photodegradation experiment) is shown in Fig. 14.

UV induced photocatalytic activity of TiO_2_-A-n% Ag composites (n = 0.5; 1; 2.5 wt%) was higher than the activity of pristine titanium dioxide (TiO_2_-A) anatase powder (see Fig. 14A). Within this group of samples, the best photocatalytic activity was observed for the sample TiO_2_-A-0.5% Ag (TiO_2_ modified with 0.5 wt% of Ag nanoparticles).

The surface modification of MM-g-CN samples with Ag NPs led to decrease in the photocatalytic activity under irradiation with UV light, whereas the photodegradation activity of silver modified MM-g-CN samples decreased with Ag content (Fig. 14B). The comparison of the extent of the AO7 degradation after 3 h long irradiation with UV light obtained for the samples from both, TiO_2_-A and MM-g-CN series (Tables 9, 10, Table S3), indicated the composite TiO_2_-A-0.5% Ag as the most photoactive.

**Table 9:** Activity and kinetic constant of pristine and Ag NPs modified TiO_2_ (UV vs. VIS lamp irradiation).

Materials	UV lamp		VIS lamp	
	Activity (%)	k_obs_ 10^−3^ (min^−1^)	Activity (%)	k_obs_ 10^−3^ (min^−1^)
TiO_2_-A	84	9.4	1	0.7
TiO_2_-A-0.5% Ag	95	24.0	10	0.8
TiO_2_-A-1% Ag	93	20.2	18	1.3
TiO_2_-A-2.5% Ag	94	21.6	38	2.5

**Table 10:** Activity and kinetic constant of pristine and Ag NPs modified MM-g-CN (UV vs. VIS lamp irradiation).

Materials	UV lamp		VIS lamp	
	Activity (%)	k_obs_ 10^−3^ (min^−1^)	Activity (%)	k_obs_ 10^−3^ (min^−1^)
MM-g-CN	94	20.3	91	10.2
MM-g-CN-0.5% Ag	94	20.2	98	17.9
MM-g-CN-1% Ag	94	18.2	98	18.8
MM-g-CN-2.5% Ag	92	14.9	96	15.2

In the case of VIS light irradiation, the pristine TiO_2_-A, as expected, did not show any photocatalytic activity and the activity increased with silver content as evident in Fig. 14C. In the case of MM-g-CN group, the highest degradation of AO7 induced by VIS light was achieved for samples MM-g-CN with 0.5 and 1 wt% Ag (Fig. 14D). The influence of Ag NPs in all samples was also expressed for 1 and 2 h of UV or VIS irradiation (see Table S3) as the extent of AO7 photodegradation, and the values ranged from 70 to 90% for both, TiO_2_-A and MM-g-CN semiconductors modified with only 0.5 wt% Ag.

Furthermore, the comparison of photodegradation activities of both semiconductor surface modified with Ag materials with recently reported studies^13,36,64–68^ are listed in Table 11, and differences in the photodegradation of acid orange 7 (AO7) or a similar methyl orange (MO) dye were shown. However, no publications were found for the Ag/melem-g-C_3_N_4_ material type (surface modification with Ag NPs); for this reason, a comparison could not be made for the photodegradation of AO7, not even for a similar molecule like MO. The innovation of this work is mainly the surface modification of Ag NPs on TiO_2_-A (anatase type) or a mixture of melem-g-C_3_N_4_ materials. Compared to our study for Ag/TiO_2_ materials, it is evident from Table 11 that the prepared materials listed in this study have a higher efficiency for the AO7 degradation, not only at UV irradiation, about 38% and eventually 35%^13^, but also at Vis irradiation, about 8%^65^. A similar trend has been observed for Ag/g-C_3_N_4_ materials for the Vis irradiation, where the photodegradation efficiency of AO7 was higher by about 50% than in the study^36^ or 10% higher if compared with results in study^68^. The positive effect on the higher photoactivity of the prepared materials of this type is probably due to the fact that it is a mixture of melem/g-C_3_N_4_. No publications were found to use these materials for UV degradation of AO7 or MO.

**Table 11:** Overview of materials used for photodegradation of AO7 or MO or AO.

Material (surface modified Ag NPs on TiO_2_ or g-C_3_N_4_)	Photodegraded dye	Condition of irradiation	Photocatalytic efficiency	Ref.
Ag/TiO_2_anatase	MO, AO	30 min and 1 h UV 300 nm	100% after 30 min and 1 h	^64^
*TiO_2_-0.1% Ag *TiO_2_-0.1–0.3% Ag	AO7	1 h UV 368 nm 1 h UV 360 nm	40% 43%	^13^
0.5% Ag/TiO_2_-rutile	MO	1 h VIS, 400 W lamp	10%	^65^
TiO_2_-Anatase-0.5% Ag TiO_2_-Anatase-2.5% Ag	AO7	1 h UV 368 nm 1 h VIS 420 nm	78% 18%	This work
Ag/g-C_3_N_4_ (1:2)	MO	1 h VIS 420 nm	10%	^36^
Ag/g-CN (4 h of melamine annealing at 550 °C)	MO	1 h VIS 420 nm	38%	^66^
Ag/g-C_3_N_4_	MO	1 h VIS 420 nm	90%	^67^
1.0%Ag/g-C_3_N_4_	MO	1 h VIS 420 nm	50%	^68^
MM-g-CN-0.5% Ag MM-g-CN-0.5% Ag	AO7	1 h UV 368 nm 1 h VIS 420 nm	71% 62%	This work

MO, Methyl Orange; AO, Orange P3R Reactive-Orange-12; AO7, Acid Orange 7,

*Mixture anatase + rutile.

The difference in the degradation rate for both TiO_2_-A and MM-g-CN series was further documented by kinetic constants. The kinetic constants (*k*_*obs*_) of all samples were calculated using the equation *ln*(*C/C*_*0*_) =  *− kt* for pseudo-first-order reaction, where *C*_0_ is the initial concentration of AO7, *C* is the concentration of AO7 at time *t* of the measurement, and *k* is the kinetic constant^69^, the obtained values are shown in Tables 9 and 10. One can observe that the determined kinetic constants for series of TiO_2_-A-n% Ag composites (n = 0.5; 1; 2.5 wt%) were in the range of 9.4–24×10^−3^ min^−1^, and the highest value was achieved for TiO_2_-A-0.5% Ag sample under UV irradiation. The achieved kinetic constants for series of MM-g-CN-n% Ag composites (n = 0.5; 1; 2.5 wt%) ranged from 14.9 to 20.3·10^−3^ min^−1^ (under UV lamp) and 10.2–18.8 × 10^−3^ min^−1^ (under VIS lamp), respectively. Two materials, pristine MM-g-CN, and MM-g-CN-0.5% Ag revealed similar values of the kinetic constants at the level > 20 × 10^−3^ min^−1^ for the irradiation performed with UV light (Table 10). Under VIS light, similar values of kinetic constants in the range 18–19 × 10^−3^ min^−1^ showed MM-g-CN samples surface modified with 0.5 and 1 wt% Ag.

**Figure 15:**
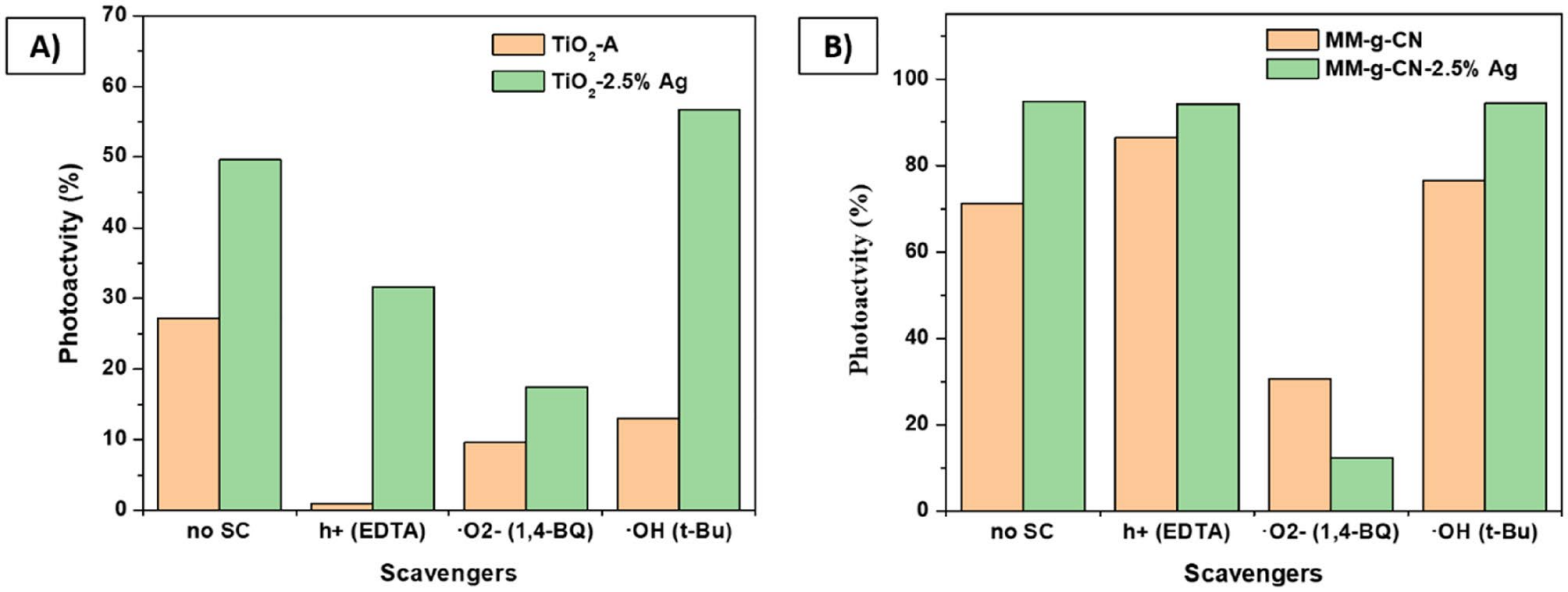
Effect of the presence of the scavengers on the photodegradation activity of: (**A**) TiO_2_-A and TiO_2_-A-2.5% Ag samples under UV irradiation and (**B**) MM-g-CN and MM-g-CN-2.5% Ag samples under VIS irradiation.

The contribution of holes, superoxide, and hydroxyl radicals was assessed by photodegradation experiments in the presence of their scavengers. In the case of the non-modified TiO_2_-A samples, the photodegradation activity was the most suppressed by the presence of the hole’s scavenger (Fig. 15A), and therefore it is assumed that the holes are the main species that participate in the photodegradation process of AO7 over non-modified TiO_2_-A (see Fig. 15A).

It has to be mentioned that the extent of the AO7 photodegradation over non-modified TiO_2_ was also suppressed in the case of the scavengers of superoxide and hydroxyl radicals but the photoactivity decrease was not so pronounced as in the case of hole scavenger. The effect of the masking of the reactive species on the photodegradation activity in the case of the TiO_2_-A-2.5% Ag sample is also evidenced in Fig. 15A. Masking of holes and superoxide radicals caused the decrease in the photodegradation activity of TiO_2_-A-2.5% Ag (Fig. 15A). In the case of this sample, the superoxide radicals show the dominant role on the photodegradation activity, on the other hand, the hydroxyl radicals had no effect on the photodegradation activity of silver modified TiO_2_-A, as evidenced in Fig. 15A. The same comparison of the effect of scavengers on the photodegradation of AO7 was performed for samples MM-g-CN and MM-g-CN-2.5% Ag (Fig. 15B). In general, the non-modified sample MM-g-CN shows a lower photodegradation activity compared to silver-modified MM-g-CN samples (compare Fig. 14B,D). For both, non-modified and silver-modified samples the superoxide radicals play the major role in AO7 photodegradation.

To investigate the stability and reusability of the best selected photocatalysts containing 0.5 wt% Ag, recycling tests of the photodegradation were performed. The TiO_2_-A-0.5% Ag sample was analyzed after 3 h under the UV (368 nm) light, and MM-g-CN-0.5% Ag sample was analyzed after 3 h under the VIS (420 nm) light. The achieved results are presented in Fig. S10. After four cycles, the photocatalytic activity of both materials remained almost at the same level (Fig. S10), what indicates good stability of their photodegradation performance.

**Figure 16:**
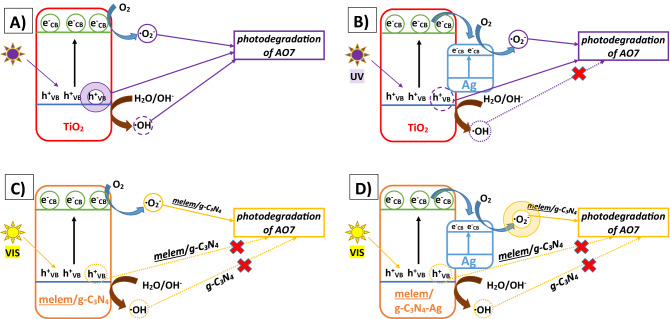
UV light induced photodegradation mechanism on (**A**) TiO_2_ NPs and (**B**) TiO_2_-Ag NPs under; VIS light induced photodegradation mechanism on (**C**) melem/g-C_3_N_4_ composites and (**D**) melem/g-C_3_N_4_-Ag composites.

The photodegradation mechanism for the given sample was derived from the photodegradation experiments in the presence of scavengers (Fig. 15) and is presented graphically in Fig. 16. For the pristine TiO_2_ NPs, all three reactive species (h^+^, ^·^O_2_^−^, ^·^OH) generated during the UV irradiation participated in the photodegradation of AO7 in the order: h^+^  > ^·^O_2_^−^ > ^·^OH (Fig. 15A). The scheme of the photodegradation process on a TiO_2_-A sample is provided in Fig. 16A, in which the holes (h^+^) as the most active species are marked by the purple double circle. ∙OH radicals are marked as the dashed purple circle in Fig. 15A. In the case of TiO_2_-Ag NPs, only ^·^O_2_^−^ and h^+^ participated in the degradation of AO7 as evidenced in Fig. 15A. The scheme of UV induced photodegradation process for TiO_2_-Ag NPs is schematically addressed in Fig. 16B.

Based on the results of the photodegradation experiments with scavengers (Fig. 15B), the photodegradation processes were proposed for pristine and silver modified MM-g-CN samples and are schematically pictured in Fig. 16C,D. In the case of both MM-g-CN and MM-g-CN-Ag, the superoxide radicals (marked by the single and double orange circles in Fig. 16D) are the only species participating in the photodegradation of AO7. Due to the lower Fermi level of Ag compared to the energy of CB of the melem/g-C_3_N_4_ composite, the photoexcited electrons in CB of g-C_3_N_4_ are transferred to Ag nanoparticles and interact with O_2_ to form the ^·^O_2_^−^ radicals. Similar behaviour was observed in Wang et al. work, where the AgI/BiSbO_4_ composite was utilized as a photocatalyst for degradation process of organic pollutants under visible light^41^. The energy necessary for the formation of ∙OH is more positive than VB of g-C_3_N_4_, and thus the holes in VB of g-C_3_N_4_ cannot react with OH^-^ to form the ∙OH radicals (marked as a dotted orange circle and arrow in Fig. 16C,D). This fact is in good agreement with the results presented in Fig. 15B, which showed that the shielding of ∙OH radicals by t-Bu did not affect the photodegradation activity of the samples in the MM-g-CN group. The same observation was also found by other authors^35,70^. Ag NPs act as an electron acceptor and inhibit the charge recombination, thus facilitating charge transfer and leading to improved photodegradation activity of the silver modified samples. This statement is in good agreement with the results presented in Fig. 15B (effect of scavengers on photodegradation activity). More ^·^O_2_^−^ radicals are generated as the Ag content in the composite increases, enhancing the photocatalytic activities of the materials in UV light (see Table S3 in the Supplementary material). The proposed schemes for MM-g-CN and MM-g-CN-Ag are aligned with previously published works^71–73^.

## Conclusions

The surface of TiO_2_ and melem/g-C_3_N_4_ materials was successfully modified with silver nanoparticles via wet, simple chemical, and low temperature method. TEM findings showed approximately 5 nm sized Ag NPs balls homogenously distributed on both modified semiconductor materials. The highest photocatalytic activity was achieved for both materials modified with a very small silver content—0.5 wt%. The photodegradation activities in excess of 95 and 94% after 3 h long irradiation with UV lamp were observed for both TiO_2_ and MM-g-CN samples with 0.5 wt% of Ag. Furthermore, the MM-g-CN sample modified with 0.5 and 1 wt% of Ag revealed the highest value of 98% after 3 h long irradiation time with VIS light. It should be also pointed here, that every modification with Ag NPs had a positive effect on both compared semiconductors under UV or VIS light processes. These results allow us to expect that the proposed low temperature chemical method which does not include the reducing agent is suitable for the surface modification of both semiconductors utilized as a photocatalyst for the degradation of model acid orange 7 dye. These investigations are in very good agreement with our previous works on surface modification with Ag NPs of anatase (TiO_2_), lithium titanium-oxide (Li_4_Ti_5_O_12_) as well as activated carbon materials which found an application in organic photovoltaics^9^, lithium-ion batteries^50^, and supercapacitors^74^, respectively.

## Supplementary Information

Supplementary Information.

## Data Availability

The data are available on the link: 10.6084/m9.figshare.21803877.
