# Supplementary material for: Effect of Ag modification on TiO2 and melem/g-C3N4 composite on photocatalytic performances
Source: Sci Rep. 2023 Mar 31;13:5270. doi: 10.1038/s41598-023-32094-6 (PMC10066401; doi:10.1038/s41598-023-32094-6)
Supplement: Supplementary file 1 — Supplementary Information. [file 41598_2023_32094_MOESM1_ESM.pdf]

Supplementary Material

Effect of Ag modification on TiO_2 and melem/g-C_3N_4 composite on photocatalytic performances

M. Michalska[1*], V. Matějka[1], J. Pavlovský[1], P. Praus[1,2], M. Ritz[1], J. Serenčíšová[3], L. Gembalová[4], M. Kormunda[5], K. Foniok[1], M. Reli[2], G. Simha Martynková[6]

[1]Department of Chemistry and Physico-Chemical Processes, Faculty of Materials Science and Technology, VŠB-Technical University of Ostrava, 17. listopadu 2172/15, 708 00 Ostrava-Poruba, Czech Republic

[2]Institute of Environmental Technology, CEET, VŠB-Technical University of Ostrava, 17. listopadu 2172/15, 708 00 Ostrava-Poruba, Czech Republic

[3]Energy Research Centre, CEET, VŠB-Technical University of Ostrava, 17. listopadu 2172/15, 708 00 Ostrava-Poruba, Czech Republic

[4]Department of Physics, Faculty of Electrical Engineering and Computer Science, VŠB-Technical University of Ostrava, 708 00 Ostrava, Czech Republic

[5]Faculty of Science, J. E. Purkyně University, Pasterurova 15, 400 96 Ústí nad Labem, Czech Republic

[6]Nanotechnology Centre, CEET, VŠB-Technical University of Ostrava, 17. listopadu 2172/15, 708 00 Ostrava-Poruba, Czech Republic

Corresponding author: monika.kinga.michalska@gmail.com (Dr. M. Michalska)

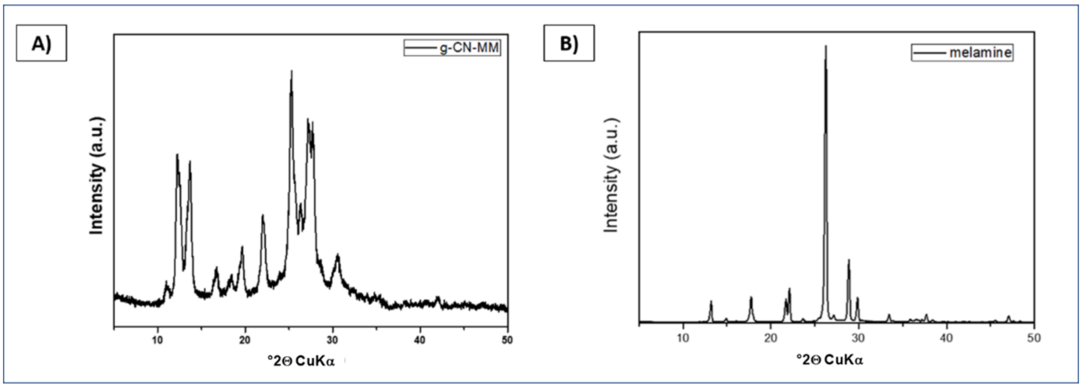

Fig. S1. XRD of (**A**) g-CN-MM and (**B**) melamine.

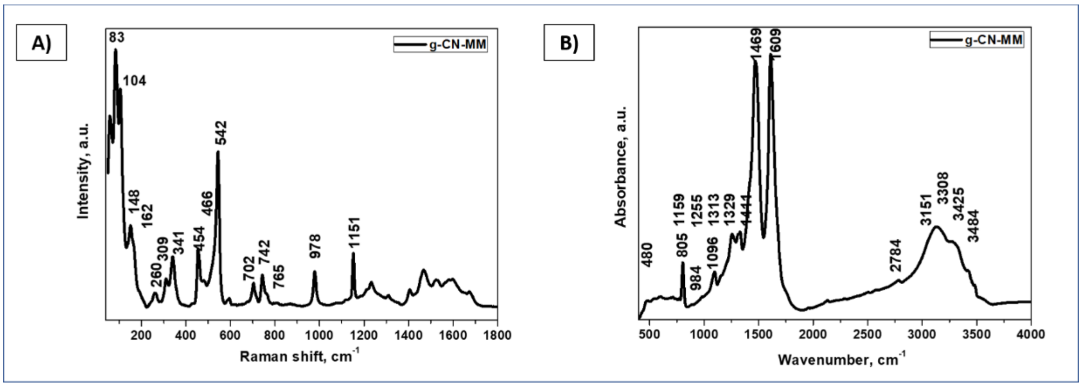

Fig. S2. The Raman (**A**) and IR (**B**) spectra of g-CN-MM powder.

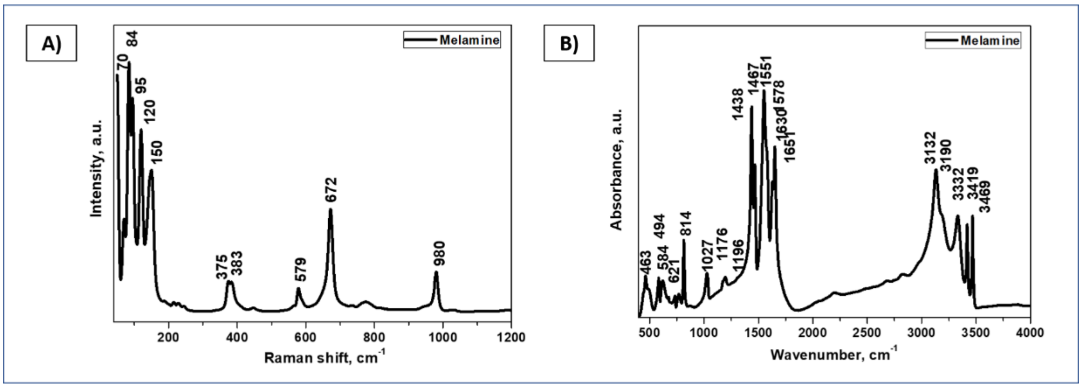

Fig. S3. The Raman (**A**) and IR (**B**) spectra of melamine powder.

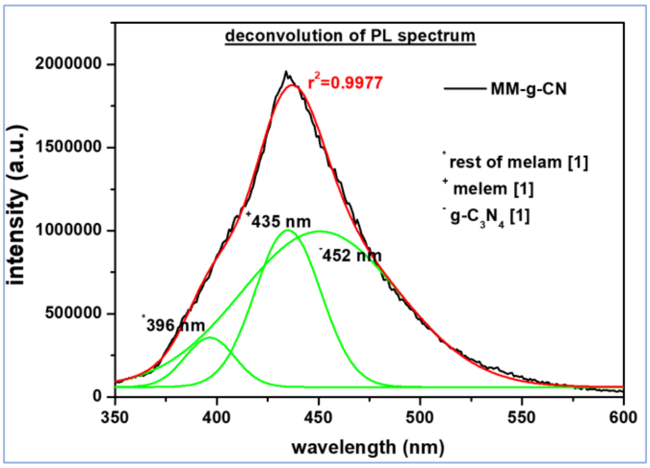

Fig. S4. PL spectra of MM-g-CN sample.

[1] Liu, N. et al. From Triazine to Heptazine: Origin of Graphitic Carbon Nitride as a Photocatalyst. ACS Omega 5, 12557-12567 (2020).

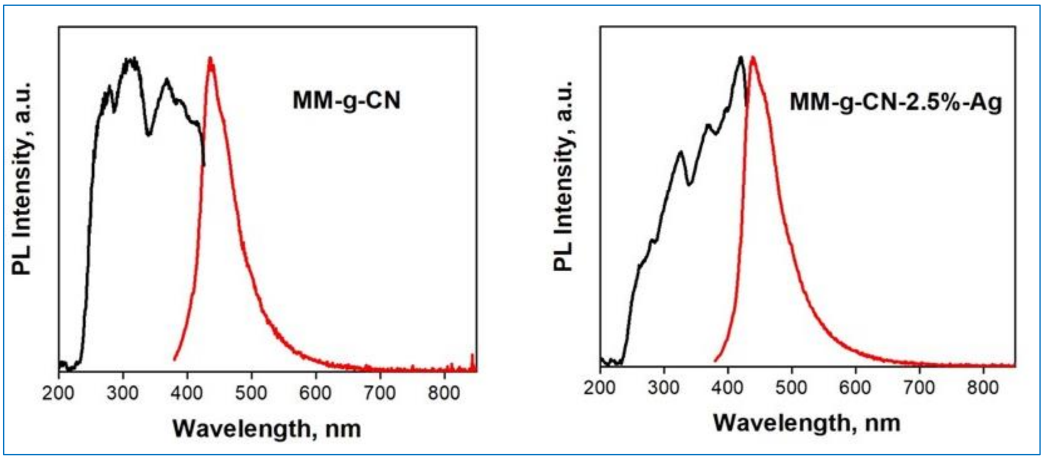

Fig. S5. PL excitation and emission spectra of MM-g-CN and MM-g-2.5% Ag.

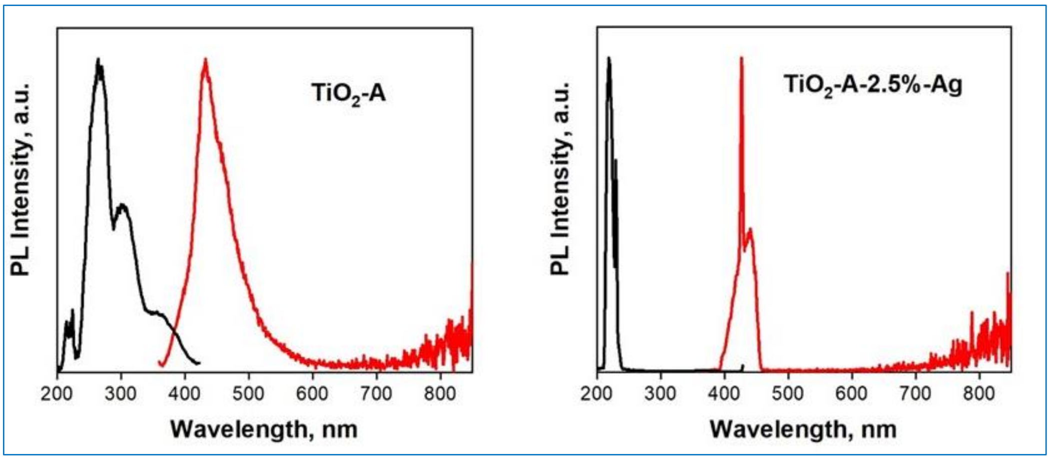

Fig. S6. PL excitation and emission spectra of TiO_2-A and TiO_2-A-2.5% Ag.

Particle distribution of TiO_2-A and MM-g-CN modified with 2.5%wt. Ag

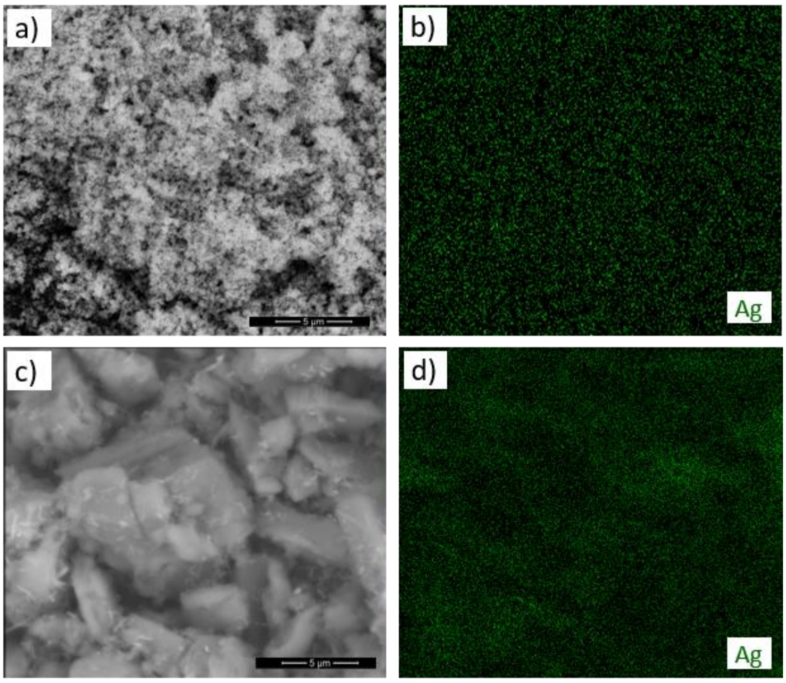

Fig. S7. Particles morphology and Ag distribution obtained for: i) TiO_2-2.5% Ag (a) and (b); ii) MM-g-CN-2.5% Ag (c) and (d).

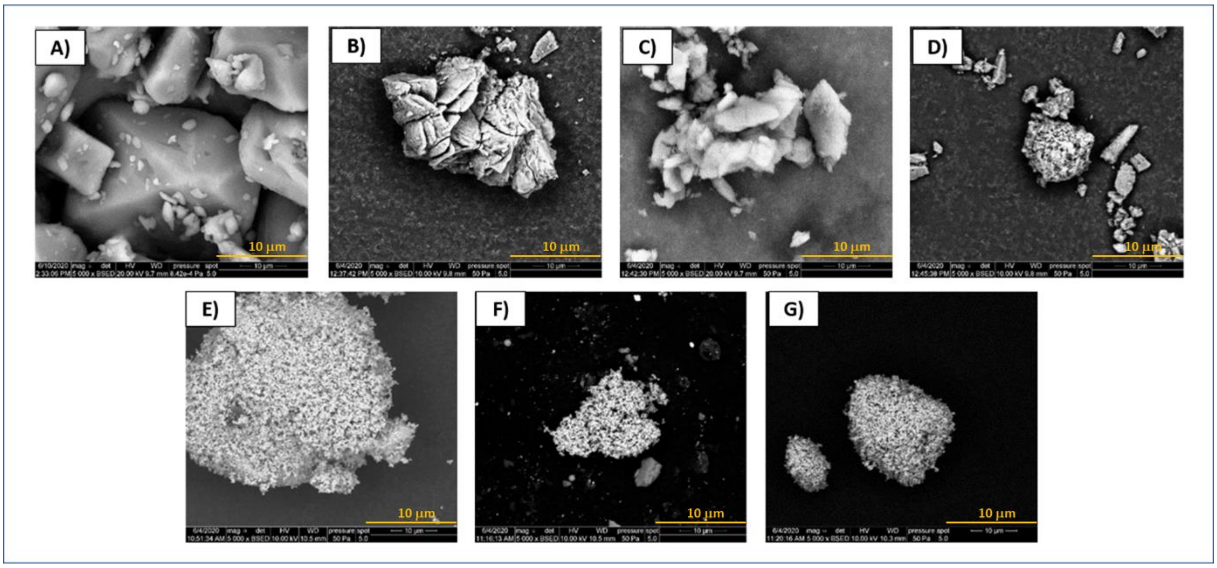

Fig. S8. SEM images of (**A**) melamine, (**B**) g-CN-MM, (**C**) MM-g-CN, (**D**) MM-g-CN-0.5% Ag, (E) TiO_2-A, (F) TiO_2-A-0.5% Ag, and (G) TiO_2-A-2.5% Ag powders at mag. 5000x, 10 µm.

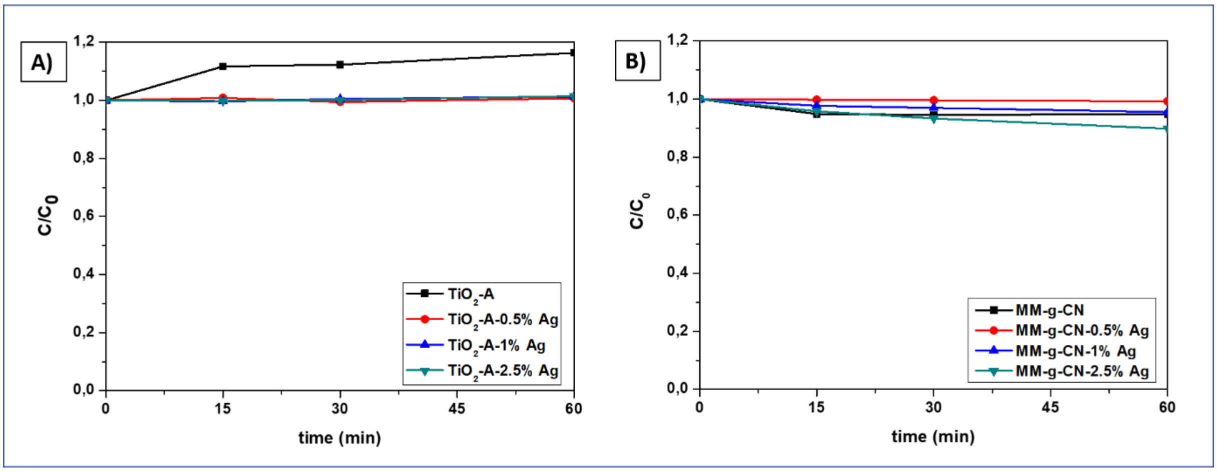

Fig. S9. Relative changes of AO7 for TiO_2-A-n% Ag (**A**) and MM-g-CN-n% Ag (**B**) powders, during the 1 h of the dark period.

Cyclic experimental data to illustrate the stability and the reusability of the materials was performed on a phenol solution. The conditions of the photodegradation experiment in each cycle were the same as for the photodegradation of AO7. For each cycle, 45 mg of photocatalyst was weighed into 150 ml of a 10 mg/L phenol solution (p.a. purity). Subsequently, the mixture was stirred in a glass container for 1 hour in the dark and then illuminated at 420 nm or 368 nm for 3 hours. After the dark period, as well as after 3 hours long irradiation period, 3 ml of suspension was taken and filtered through a 0.20 μm pore size syringe filter (CHROMAFIL GF/RC-20/25 filters, Macherey-Nagel, Germany).

The determination of the concentration of the phenol in the filtrate after individual cycles (after dark as well as the irradiation period of each cycle) was carried out as follows. A 1 ml sample of the filtrate was taken and added to 1 ml of solution A (solution A: 0.4717 mol/L Na_2CO_3, p.a. purity), then 1 ml of a mixture of solution B+C (0.025 ml B solution to 5 ml C solution) was added, where solution B was prepared as follows: 0.6900 g of p-nitroaniline (p.a. purity) in 155 ml of HCl (36 %, p.a. purity) (1:9) and then added to a 1-liter volumetric flask and topped up with distilled water; and solution C was prepared as follows: $NaNO_2$ (p.a. purity) aqueous solution with a concentration of 12.175 mol/L. After 15 minutes of colouring (brown-yellow coloration), the absorbance was measured at 470 nm. The suspensions were subsequently filtered and dried at 100 °C to a constant weight. Subsequently, filter cakes were ground and homogenized in an agate mortar and the samples were weighed again (45 mg) for the next cycle and the procedure was repeated identically. In this way, the 4 cycles were performed with a constant amount of photocatalyst. The studies were performed for the selected materials with 0.5 wt.% Ag catalysts.

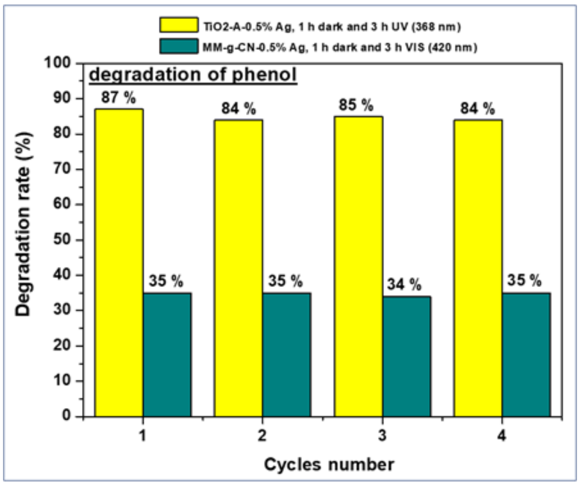

Fig. S10. The reusability of phenol under UV light for TiO_2-A-0.5% Ag and VIS light for MM-g-CN-0.5% Ag materials.

Table S1. The band assignment of spectra of melamine.

**Raman**	**Assignment**
**980 cm^{-1}**	deformation vibration of triazine ring (in plane)
**729 cm^{-1}**	deformation vibration of triazine ring (out of plane)
**579 cm^{-1}**	deformation vibration of triazine ring (out of plane)
**383 cm^{-1}**	deformation vibration of triazine ring (out of plane)
**375 cm^{-1}**	
**150 cm^{-1}**	lattice vibration of melamine
**120 cm^{-1}**	lattice vibration of melamine
**95 cm^{-1}**	lattice vibration of melamine
**84 cm^{-1}**	lattice vibration of melamine
**70 cm^{-1}**	lattice vibration of melamine
**IR**	**Assignment**
**3469 cm^{-1}**	asymmetric stretching vibration of $-NH_2$
**3419 cm^{-1}**	symmetric stretching vibration of $-NH_2$
**3332 cm^{-1}**	stretching vibration of N-H
**3190 cm^{-1}**	combination band of deformation vibration of $-NH_2$ and stretching vibration of C-N
**3132 cm^{-1}**	stretching vibration of N-H
**1651 cm^{-1}**	deformation vibration of $-NH_2$
**1630 cm^{-1}**	deformation vibration of $-NH_2$
**1578 cm^{-1}**	stretching vibration of triazine ring
**1551 cm^{-1}**	stretching vibration of triazine ring
**1467 cm^{-1}**	symmetric stretching (breathing) vibration of triazine ring
**1438 cm^{-1}**	stretching vibration of triazine ring
**1027 cm^{-1}**	deformation vibration of triazine ring (in plane)
**814 cm^{-1}**	deformation vibration of triazine ring (out of plane)

Table S2. X^2 values of fitting PL decay curves with various models

Material	1st order (X^2)	2nd order (X^2)	Stretched (X^2)	3rd order (X^2)
MM-g-CN	12.94	1.447	1.803	0.976
MM-g-CN-2.5% Ag	14.52	1.650	2.14	1.017
TiO_2-A	15.46	1.740	1.884	1.001

Table S3. Activity of pristine and modified samples with Ag NPs after 1 and 2 hours of irradiation with UV and VIS light.

	**UV lamp**		**VIS lamp**	
**Materials**	**Activity (%) after 1 h**	**Activity (%) after 2 h**	**Activity (%) after 1 h**	**Activity (%) after 2 h**
TiO_2-A	42	75	0	0
TiO_2-A-0.5% Ag	78	91	4	5
TiO_2-A-1% Ag	73	88	1	4
TiO_2-A-2.5% Ag	76	90	18	26
MM-g-CN	73	88	41	71
MM-g-CN-0.5% Ag	71	89	62	91
MM-g-CN-1% Ag	65	87	63	93
MM-g-CN-2.5% Ag	56	83	88	96
